# The coral *Acropora loripes* genome reveals an alternative pathway for cysteine biosynthesis in animals 

**DOI:** 10.1126/sciadv.abq0304

**Published:** 2022-09-23

**Authors:** Octavio R. Salazar, Prasanna N. Arun, Guoxin Cui, Line K. Bay, Madeleine J. H. van Oppen, Nicole S. Webster, Manuel Aranda

**Affiliations:** ^1^ Marine Science Program, Biological and Environmental Sciences and Engineering Division, King Abdullah University of Science and Technology (KAUST), Thuwal 23955-6900, Kingdom of Saudi Arabia.; ^2^ Red Sea Research Center, King Abdullah University of Science and Technology, Thuwal, Kingdom of Saudi Arabia.; ^3^ Australian Institute of Marine Science, Townsville, Queensland, Australia.; ^4^ AIMS@JCU, Division of Research and Innovation, James Cook University, Townsville, Australia.; ^5^ School of BioSciences, University of Melbourne, Parkville, Victoria 3010, Australia.; ^6^ Australian Centre for Ecogenomics, University of Queensland, St Lucia, Australia.; ^7^ Australian Antarctic Division, Department of Agriculture, Water and the Environment, Kingston, Australia.

## Abstract

The metabolic capabilities of animals have been derived from well-studied model organisms and are generally considered to be well understood. In animals, cysteine is an important amino acid thought to be exclusively synthesized through the transsulfuration pathway. Corals of the genus *Acropora* have lost cystathionine β-synthase, a key enzyme of the transsulfuration pathway, and it was proposed that *Acropora* relies on the symbiosis with dinoflagellates of the family Symbiodiniaceae for the acquisition of cysteine. Here, we identify the existence of an alternative pathway for cysteine biosynthesis in animals through the analysis of the genome of the coral *Acropora loripes*. We demonstrate that these coral proteins are functional and synthesize cysteine in vivo, exhibiting previously unrecognized metabolic capabilities of animals. This pathway is also present in most animals but absent in mammals, arthropods, and nematodes, precisely the groups where most of the animal model organisms belong to, highlighting the risks of generalizing findings from model organisms.

## Teaser

Exploration of the genome of the nonmodel organism *Acropora loripes* reveals unrecognized metabolic capabilities of animals.

## INTRODUCTION

Cysteine is one of two sulfur-containing amino acids, together with methionine, and is required for proper development, sulfur metabolism, and production of antioxidants. In animals, cysteine biosynthesis is considered to be exclusively carried out through the transsulfuration pathway (*1*), which relies on cystathionine β-synthase (CBS) and pyridoxal 5′-phosphate (PLP) (vitamin B_6_) as a cofactor to catalyze the condensation of homocysteine and serine to produce cystathionine. Cystathionine is subsequently converted into l-cysteine by cystathionine γ-lyase. In humans, mutations in *CBS* lead to homocystinuria (*2*), a disease caused by the accumulation of homocysteine, resulting in disorders such as mental retardation, skeletal abnormalities, optic lens dislocation, and cardiovascular disease (*3*). CBS is not present in corals of the genus *Acropora* (*4*, *5*), and species within this genus might instead rely on their symbiosis with dinoflagellates of the family Symbiodiniaceae for the acquisition of cysteine (*4*). Cysteine is required for the production of glutathione, an important and crucial antioxidant for the response to reactive oxygen species generated by the symbiont under heat stress. It was, therefore, proposed that the reliance on symbiont-derived cysteine may contribute to the increased susceptibility of *Acropora* species to thermal bleaching compared to many other coral genera (*6*, *7*).

Plants and bacteria use a different pathway for cysteine biosynthesis in which cysteine is synthesized through the sulfate assimilation pathway, where l-serine is converted into *O*-acetyl-l-serine by a serine *O*-acetyltransferase and then converted into l-cysteine by an *O*-acetylserine sulfhydrylase (CysK/CysM) (*8*, *9*). Analysis of cysteine biosynthesis pathways in Kyoto Encyclopedia of Genes and Genomes (KEGG) (*10*, *11*) shows that metazoans lack the required enzymes for cysteine biosynthesis through the sulfate assimilation pathway. However, a recently discovered pathway for cysteine biosynthesis, not yet included in enzymatic reaction databases such as MetaCyc (*12*) or KEGG, has been identified in fungi (*13*), in which l-serine is converted into *O*-succinyl-l-serine by an l-serine-*O*-succinyltransferase (Cys2) and then into l-cysteine by *O*-succinylserine sulfhydrylase (Cys1a). Mutations in either *Cys2* or *Cys1a* lead to cysteine auxotrophy in *Schizosaccharomyces pombe* (*14*–*16*).

Here, we explored the genome of the coral *Acropora loripes* for alternative cysteine biosynthesis pathways in animals. We show that the loss of the *CBS* gene in *Acropora* corals was likely due to the activity of transposable elements. We identified the presence of an alternative cysteine biosynthesis pathway through the action of homologs of the proteins Cys2 and Cys1a, previously only described in fungi. Moreover, this alternative pathway was found throughout the animal kingdom except for vertebrates, arthropods, and nematodes and was also found in other eukaryotes except for higher plants. We further show that the Cys1a and Cys2 proteins of *A. loripes* can complement the *Cys1a*Δ and *Cys2*Δ mutants in *S. pombe*, demonstrating that the coral proteins can synthesize cysteine through this alternative pathway and revealing a previously unrecognized pathway for cysteine biosynthesis in animals.

## RESULTS

### CBS loss in the genus *Acropora*

**Fig. 1.**
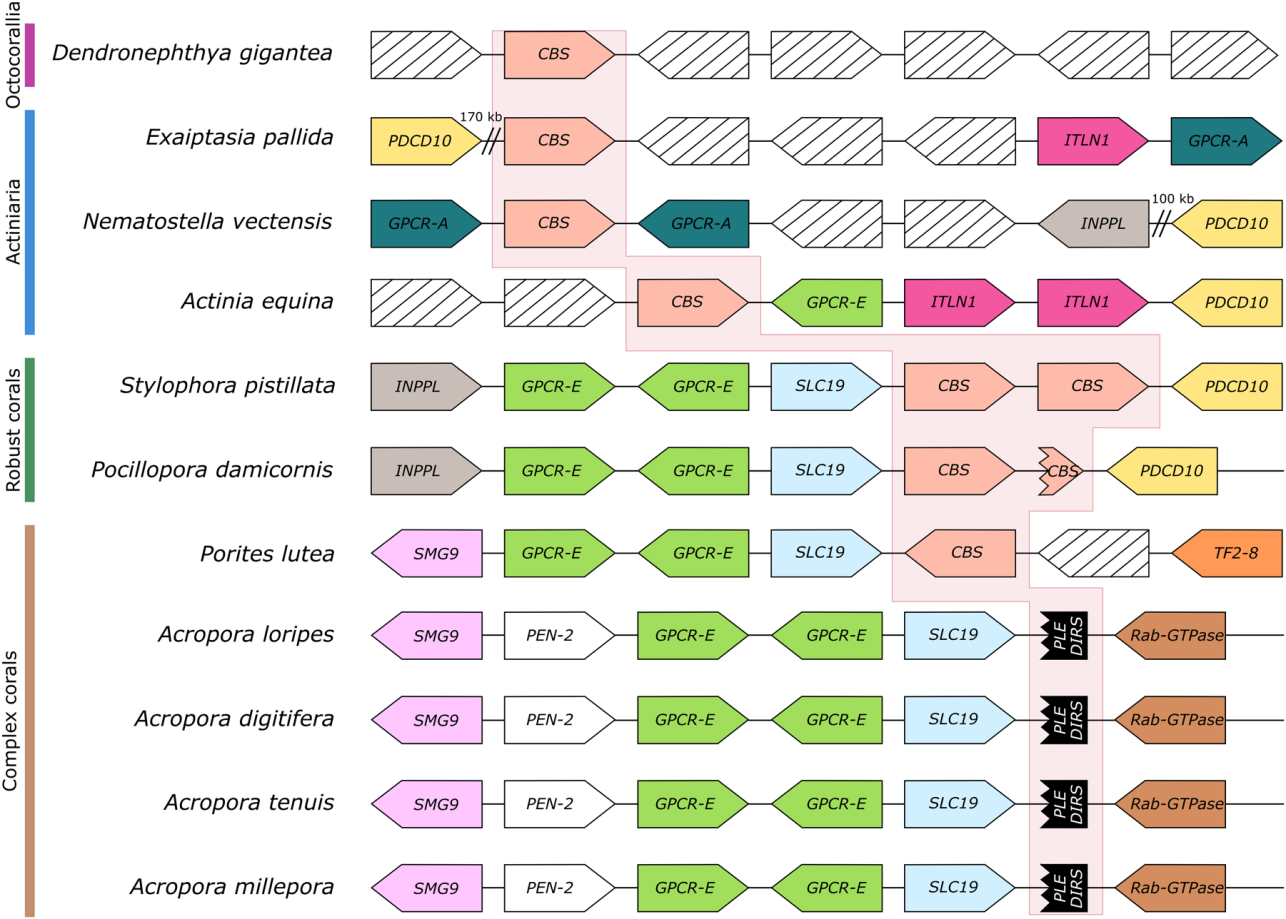
*CBS* gene loss in the genus *Acropora*. Gene synteny at the *CBS* gene locus across anthozoans. Shape tip depicts gene orientation. The red background highlights the *CBS* locus. Homologous genes are depicted with the same color. *CBS* in red, programmed cell death protein 10 (*PDCD10*) in yellow, intelectin-1 (*ITLN1*) in magenta, phosphatidylinositol 3,4,5-trisphosphate 5-phosphatase 2 (*INPPL*) in gray, G protein–coupled receptor class A (*GPCR-A*) in teal, G protein–coupled receptor class E (*GPCR-E*) in green, thiamine transporter (*SLC19*) in light blue, protein SMG9 (*SMG9*) in lilac, transposon Tf2-8 polyprotein (*TF28*) in orange, gamma-secretase subunit pen-2 (*PEN*-2) in white, Penelope-like repeat (*PLE*) and *Dictyostelium* intermediate repeat sequence (*DIRS*) in black, Rab-GTPase (guanosine triphosphatase) (*Rab-GTPase*) in brown, and genes without conservation across species in black stripes.

To analyze the loss of CBS in *Acropora*, we first sequenced the genome of *A. loripes*, resulting in an assembly composing of 335 scaffolds and a BUSCO (Benchmarking Universal Single-Copy Orthologs) (*17*) score against the Metazoa dataset v.10 of: complete, 96.1%; fragmented, 1.7%; and missing, 2.2% (Table 1, figs. S1 to S4, tables S1 to S4, and data S1), being one of the most complete and least fragmented coral genomes to date. The *CBS* locus was examined in different Anthozoa species, revealing conserved synteny across species that is traceable across the evolutionary tree (Fig. 1). To rule out synteny misinterpretation due to biases in gene prediction, we analyzed syntenic genes surrounding the *CBS* locus in all stony coral genomes using BLAST (*18*) to ensure that missing genes were truly missing in the vicinity of that locus of the corresponding genome. A duplication of *CBS* seems to have occurred in robust corals, as two copies are found in tandem duplication in *Stylophora pistillata* and *Pocillopora damicornis*, albeit the latter showing a disruption of the extra copy (Fig. 1). A high degree of synteny can be observed within stony corals, where *CBS* is preceded by a thiamine transporter (*SLC19A3*) and two facing copies of G protein–coupled receptor class E (*GPCR*-*E*) (Fig. 1). A region of ~17 kb, devoid of any genes, is found in all *Acropora* species at the location where *CBS* would be expected to occur. In this region, scars of two transposable elements were found: A *Dictyostelium* intermediate repeat sequence (*DIRS*) type (*19*) and a *Penelope*-like element (*PLE*) (*20*). These remnants suggest that the transposition of these elements might have caused the loss of *CBS* in *Acropora*. Nevertheless, other uncharacterized repetitive elements were also found in this region and cannot be ruled out as the cause of *CBS* loss. A retrotransposon Tf2-8 polyprotein (*TF2-8*) was found close to the *CBS* gene in *Porites lutea*, a member of complex corals together with *Acropora*. The appearance of *TF2-8* at the *CBS* locus is accompanied by an inversion of the *CBS* gene and a loss in synteny (Fig. 1). This synteny break is only found in complex corals and indicates a locus instability in their common ancestor. TF2s are long terminal repeat retrotransposons that form virus-like structures from a single transcript and primarily move through recombination with preexisting copies of other *TF2* genes (*21*). It is possible that the insertion and further movement of this retrotransposon led to the loss of *CBS* in the genus *Acropora*.

**Table 1.** Genome assembly statistics of *A. loripes* compared to other sequenced *Acropora* species used in this study.

**Parameter**	***Acropora loripes* (this study)**	***Acropora digitifera* (*4*)**	***Acropora tenuis* (*62*)**	***Acropora millepora* (*64*)**
Assembly size of scaffolds (Mb)	401.87	447.49	486.81	386.60
Assembly size of contigs (Mb)	401.86	379.28	486.81	349.00
Number of scaffolds/contigs	335/398	2,421/54,033	614/614	3,869/20,440
Scaffold N50	2.82 Mb	483.55 kb	2.83 Mb	494.53 kb
Contig N50	2.35 Mb	11.00 kb	2.83 Mb	36.67 kb
Repetitive elements (%)	43.13	30.99*	43.39*	34.55
GC content (%)	38.99	39.04	39.07	38.85
Gap content (Ns) (%)	0.002	15.24	0	9.72
Number of predicted genes	27,152	33,865	30,327	26,615
Completeness by BUSCO (Metazoa) C:F:M (%)^†^	96.1:1.7:2.2	77.5:10.2:12.3	88.6:4.8:6.6	88.0:6.5:5.5

*Values generated by de novo masking genomes from other studies.

†Percentages of complete, fragmented, and missing BUSCO genes on predicted proteins per genome against the Metazoa v.10 dataset.

### Cys1a is a previously unrecognized cysteine synthase in animals

**Fig. 2.**
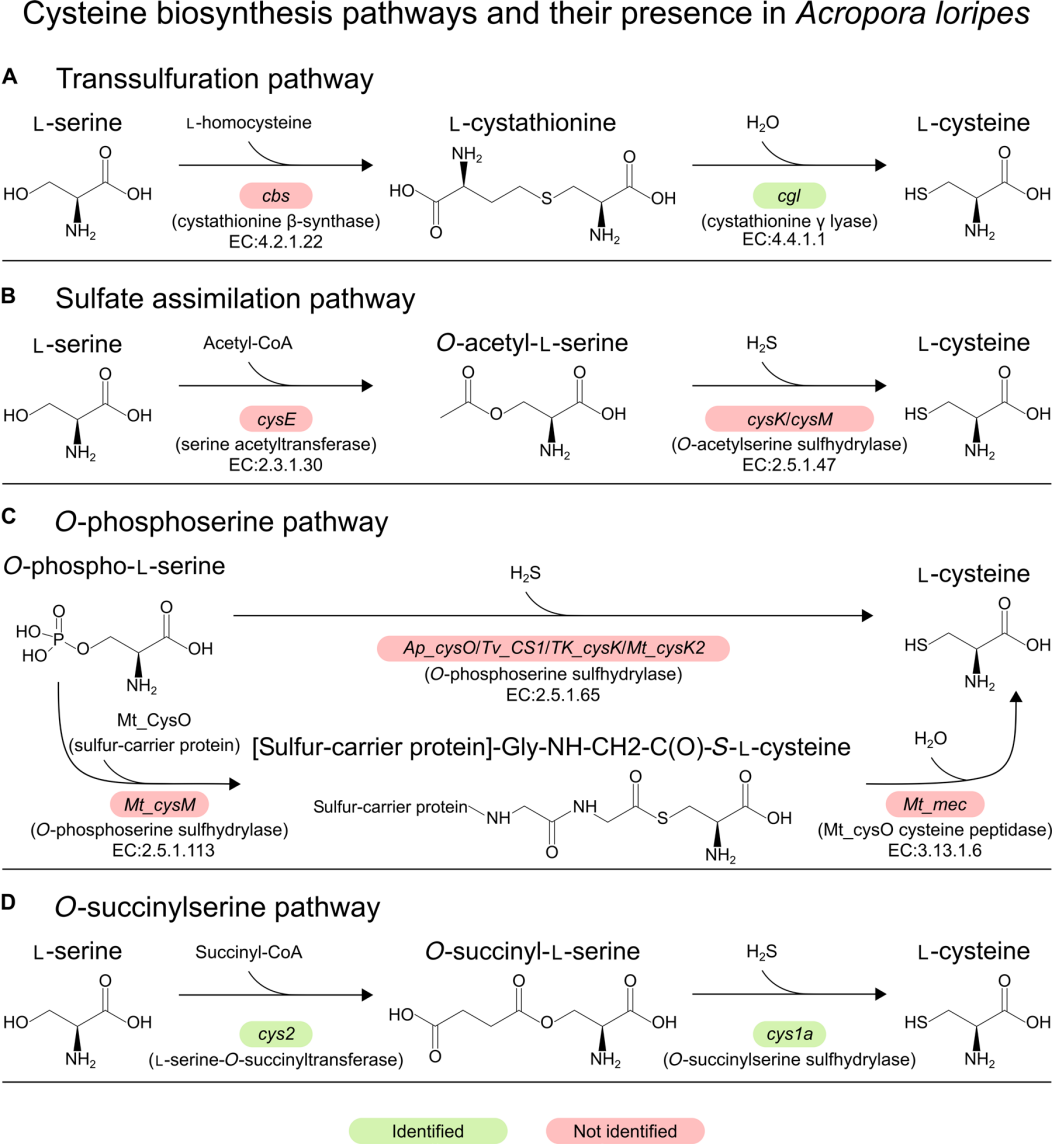
Cysteine biosynthesis pathways and the identification of their genes in *A. loripes*. (**A**) Transsulfuration pathway. (**B**) Sulfate assimilation pathway. (**C**) *O*-phosphoserine pathway. (**D**) *O*-succinylserine pathway. Genes are denoted by colored boxes: Red not identified and green identified in the genome of *A. loripes*. EC (Enzyme Commission) numbers are included for the available classified reactions.

**Fig. 3.**
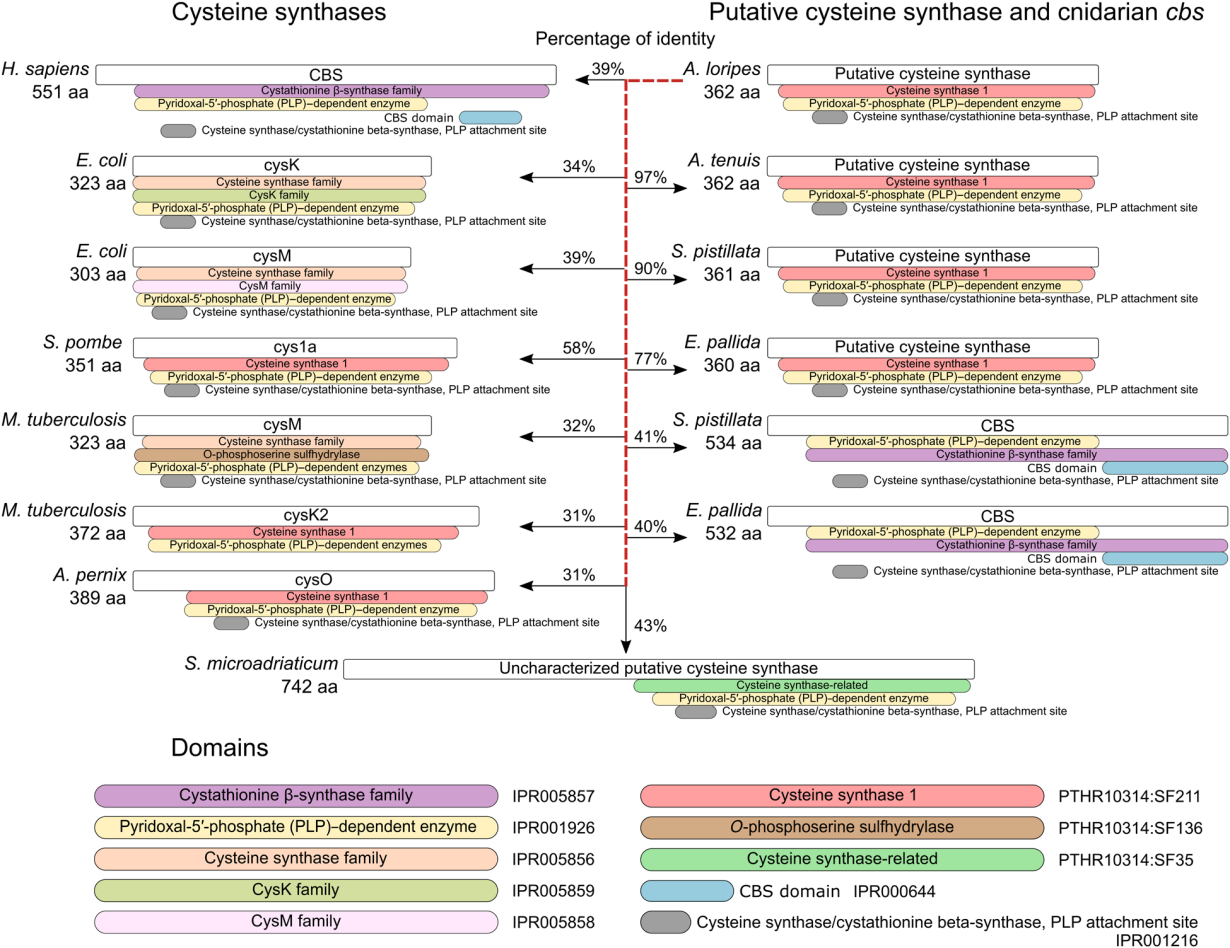
Cysteine synthases and their domain architectures. Sequence identities are shown between *A. loripes* putative cysteine synthase and other cysteine synthases. Protein domains are identified by their InterPro or PANTHER domains.

Next, we analyzed the encoded protein domains in the genome of *A. loripes* for proteins involved in cysteine biosynthesis (Fig. 2) and identified the existence of a gene (*alor_g9760*), from now on referred to as *Acys1a* for *Acropora Cys1a*, encoding for a protein of 362 amino acids with a cysteine synthase 1 domain (PTHR10314:SF211). This gene also encodes a pyridoxal-5′ phosphate–dependent enzyme domain (IPR001926) and a pyridoxal-phosphate attachment site (IPR001216), both of which are common to all cysteine synthases (Fig. 3). *Acys1a* contains introns, was identified in all previously analyzed anthozoans, and shows low percentages of identity against cysteine synthases from the canonical pathways (Fig. 3), indicating that this gene did not arise from contamination and implying a conserved function rather than a remnant of a lost pathway. Acys1a protein sequence exhibits 58% of identity against *S. pombe* Cys1a, being the highest among cysteine synthases. Furthermore, they share the same domain architecture (Fig. 3).

**Fig. 4.**
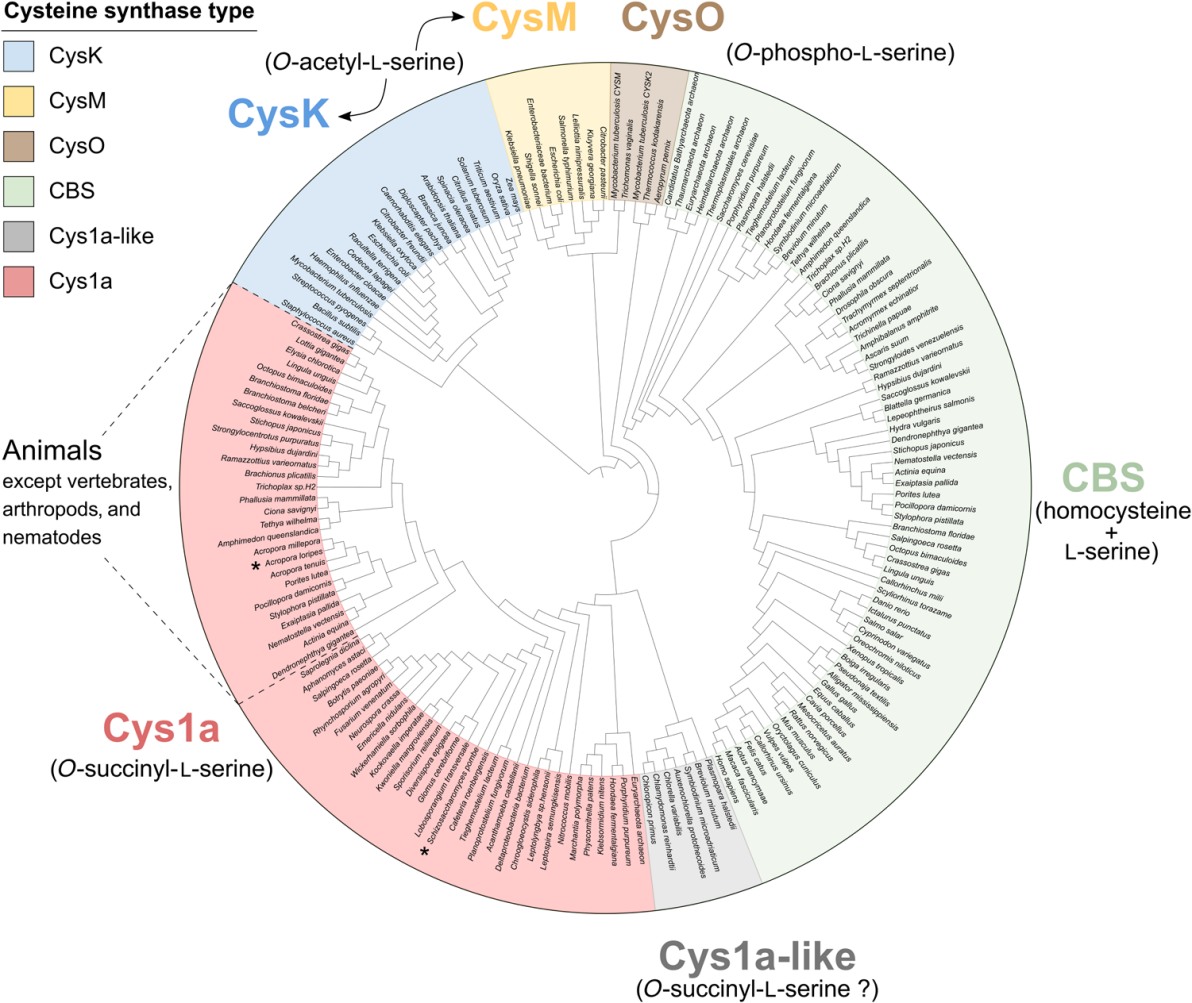
Phylogenetic reconstruction of cysteine synthases. Protein sequences from cysteine synthases belonging to the transsulfuration pathway (CBS), sulfate assimilation pathway (CysK and CysM), *O*-phosphoserine pathway (CysO), and *O*-succinylserine pathway (Cys1a) were used for the generation of a phylogenetic tree. Primary substrates for each cysteine synthase are shown in parentheses. Sequences similar to *A. loripes* putative cysteine synthase Acys1a and *S. pombe* Cys1a (both denoted by *) were included in the analysis and either grouped within the Cys1a cluster or formed a sister clade classified as Cys1a-like. Acys1a was classified as a Cys1a-type cysteine synthase together with animal sequences from all lineages except for vertebrates, arthropods, and nematodes. The phylogenetic tree was generated with IQ-TREE (*77*) and visualized with iTOL (*79*).

To classify Acys1a, we performed a phylogenetic analysis with similar sequences, including protein sequences similar to *S. pombe* Cys1a, as well as sequences from other cysteine biosynthesis pathways (data S3). A phylogenetic tree was reconstructed, classifying cysteine synthases into six main groups (Fig. 4): the CBS group, from the transsulfuration pathway; CysK and CysM groups, from the sulfate assimilation pathway; CysO, from the *O*-phosphoserine pathway (*22*–*26*); Cys1a, from the succinylserine pathway; and the unknown group, greatly resembling Cys1a proteins. Acys1a fell within the Cys1a group and formed a subgroup composed of sequences from species across all metazoan lineages except for vertebrates, arthropods, and nematodes. Search for homologous proteins in these three metazoan groups in UniProt (*27*) revealed that Cys1a is absent in them. Only one homologous protein was identified in the nematode *Necator americanus*, but closer inspection revealed that the protein belonged to a very small scaffold with two genes, both devoid of introns and showing 100% identity to proteins from *Paraburkholderia silvatlantica*, indicating that these proteins might stem from bacterial contamination in the genome assembly. Cys1a proteins were also present in nonmetazoan eukaryotes, except for higher plants (Fig. 4). Furthermore, they were identified in bacteria and archaea. A group of proteins greatly resembling Cys1a formed an independent clade, which we called Cys1a-like. Proteins of the group Cys1a-like have the cysteine synthase–related domain (PTHR10314:SF35) instead of the cysteine synthase 1 domain (PTHR10314:SF211) found in Cys1a (Fig. 3). Proteins from the coral symbionts of the family Symbiodiniaceae fall within this clade, but these organisms also have the *CBS* gene.

### Cys2 is also present throughout metazoans

Cys1a requires *O*-succinyl-l-serine for the biosynthesis of cysteine, which, in the fungi *S. pombe*, is provided by the l-serine *O*-succinyltransferase (SST) Cys2 through the transfer of a succinyl group from succinyl-CoA (coenzyme A) to l-serine (Fig. 2) (*13*). We screened the *A. loripes* genome for homologous proteins of Cys2 from *S. pombe* and identified a gene (*alor_g16104*), subsequently referred to as *Acys2*, encoding for a putative Cys2 protein of 465 amino acids. Cys2 belongs to the MetX family of enzymes, previously thought to only be l-homoserine *O*-acetyltransferases (HATs). These enzymes are crucial for the biosynthesis of methionine (*28*) but were recently shown to also function as l-homoserine *O*-succinyltransferases (HSTs), l-serine *O*-acetyltransferases (SATs), and SSTs (*13*, *29*). Cys2 enzymes have been shown to be primarily SSTs and have almost 300 times greater efficiency with l-serine than with l-homoserine (*13*); furthermore, Cys2 mutants of *S. pombe* become cysteine auxotrophic (*14*, *15*). However, because of the recent discovery of the different activities of MetX proteins, it has been estimated that more than 60% of the MetX proteins are incorrectly annotated (*13*), rendering the classification of MetX proteins through the current annotation from homologous proteins ineffective. MetX proteins have been divided into six groups according to their primary enzymatic activities (*13*): MetX-G1, composed of HATs; MetX-G2, with HSTs; MetX-G3a, with HSTs and SSTs; MetX-G3b, with HSTs; MetX-G3c, with SSTs; and MetX-G4, proteins without any detected enzymatic activity. *S. pombe* Cys2 is classified as a MetX-G3c.

**Fig. 5.**
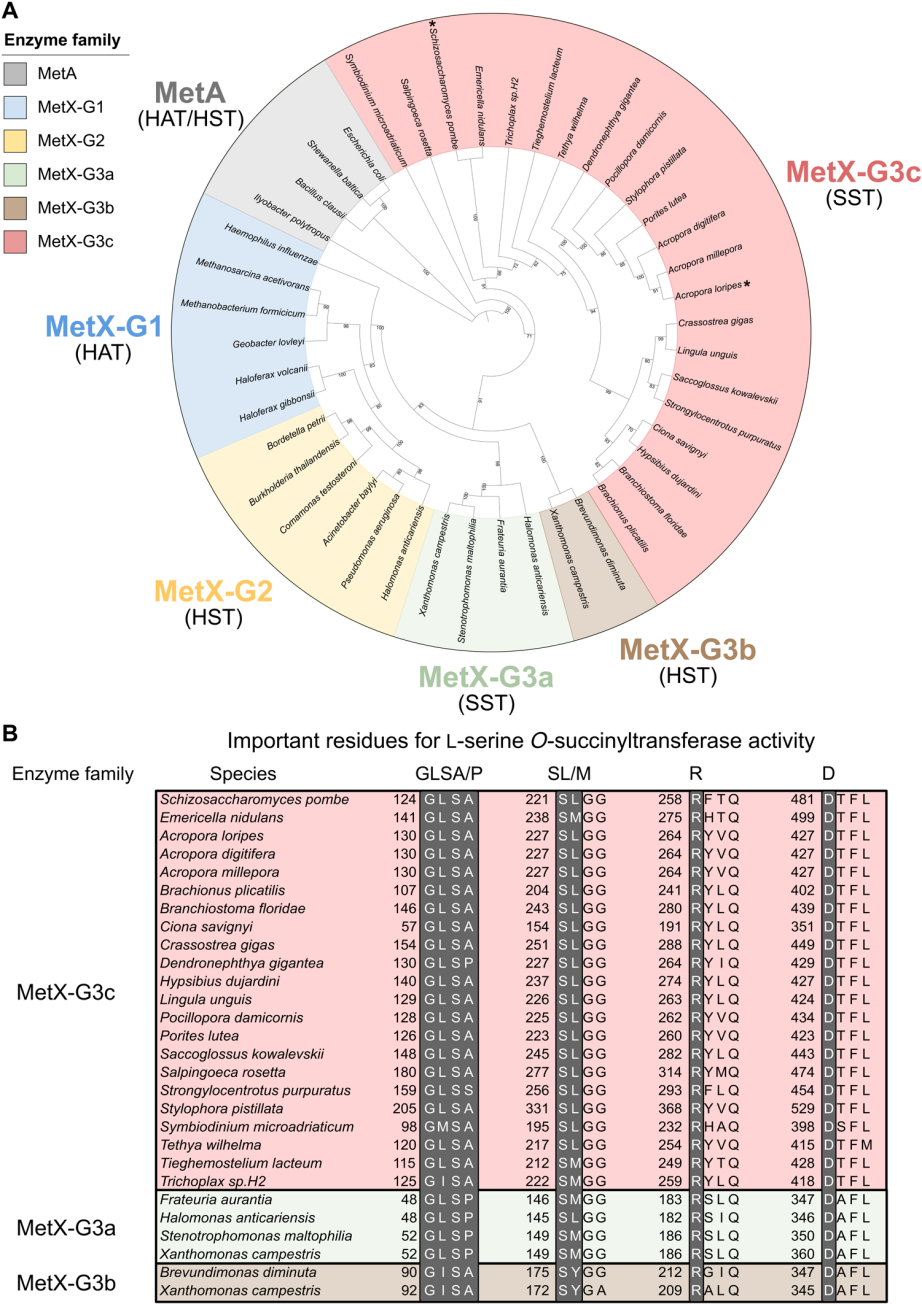
Classification of MetX proteins. (**A**) Phylogenetic reconstruction of MetX proteins: MetX-G1 in blue, MetX-G2 in yellow, MetX-G3a in green, MetX-G3b in brown, MetX-G3c in red, and MetA as the outgroup in gray. Each group has its enzymatic activity in parenthesis: HAT, HST, and SST. *A. loripes* putative Cys2 (Acys2) and *S. pombe* Cys2 are denoted by an asterisk (*). Acys2 was classified as a MetX-3Gc SST together with animal sequences from all lineages except for vertebrates, arthropods, and nematodes. The phylogenetic tree was generated with IQ-TREE and visualized with iTOL. (**B**) Identification of important residues in putative Cys2 protein sequences for SST activity. MetX-G3c in red, MetX-G3a in green, and MetX-G3b in brown. Important residues for an SST activity are highlighted in gray. *A. loripes* Cys2 and other eukaryotic putative Cys2 proteins have all the required residues for SST activity.

To classify the Acys2 protein into a MetX group and avoid possible previous misannotations, we retrieved sequences of experimentally validated MetX proteins (*13*) and sequences from all eukaryotic lineages with similarities to Acys2 and *S. pombe* Cys2 (data S3). We then reconstructed a phylogenetic tree with MetA proteins as the outgroup, a family of proteins with similar activities to MetX but not genetically related. The tree was resolved into six groups in line with their enzymatic activities (Fig. 5A). Acys2 fell within the MetX-G3c group and, similarly to Cys1a, formed a subgroup with other metazoans. Furthermore, no MetX-G3c sequences from vertebrates, arthropods, or nematodes were identified. A number of key residues have been previously identified in MetX-G3 proteins required for their SST activity (*13*, *30*): Gly-Leu-Ser-Ala/Pro and the similar Gly-Ile-Ser-Ala at positions 52 to 55 based on *Xanthomonas campestris* MetX-3Ga; Ser-Leu/Met at positions 149 to 150; Arg^183^ based on *Frateuria aurantia* MetX-3Ga; and Asp^360^ based on *X. campestris* MetX-3Ga. Furthermore, it is considered that having Ser-Tyr instead of Ser-Leu/Met at positions 149 to 150 results in l-homoserine specificity instead of l-serine (*13*), as observed in MetX-3Gb enzymes. Acys2 contains all these important residues for SST activity and was thus classified as a MetX-3Gc enzyme (Fig. 5B). Moreover, putative Cys2 proteins from animals and other eukaryotic lineages also contain these residues (Fig. 5B).

### *A. loripes* Cys1a and Cys2 proteins rescue cysteine biosynthesis in vivo

**Fig. 6.**
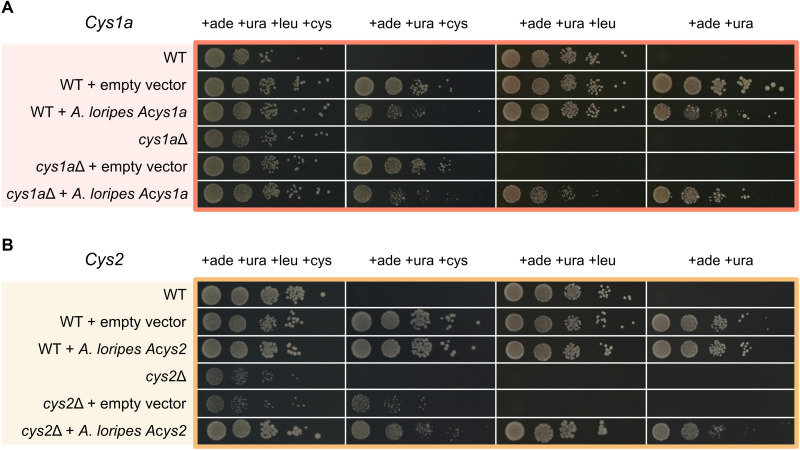
Involvement of *A. loripes* Cys1 and Cys2 proteins in cysteine biosynthesis in vivo. Yeast spot assays in *S. pombe* mutants grown in the presence or absence of l-cysteine for l-cysteine auxotrophy gene complementation with l-leucine as selection marker. (**A**) *A. loripes Cys1a* gene (*Acys1a*) complementation assay. (**B**) *A. loripes Cys2* gene (*Acys2*) complementation assay. Strains: WT (ED666), *ade6 ura4 leu1*; *cys1a*Δ, *ade6 ura4 leu1 cys1a*::*kanMX4*; and *cys2*Δ, *ade6 ura4 leu1 cys2*::*kanMX4*. All assays were carried out with the addition of adenine and uracil as the background strain (WT) is auxotrophic for both. The plasmid pREP3xN, containing *LEU2* as selection marker to complement leucine auxotrophy in the ED666 WT strain, was used as the expression vector and transformed empty, with *A. loripes Acys1a* gene or with *A. loripes Acys2* gene. All assays were grown in EMM medium and done in triplicates, with the figure showing one representative assay.

To confirm that the animal homologs of Cys1a and Cys2 can indeed catalyze the biosynthesis of cysteine, we transformed codon-optimized versions of the *A. loripes* genes *Acys1a* and *Acys2* into the cysteine auxotrophic mutants of *Cys1a*Δ and *Cys2*Δ in *S. pombe* (*31*), respectively. Similarly to *A. loripes*, *S. pombe* does not have the CBS enzyme from the transsulfuration pathway (*32*) and relies solely on the *O*-succinylserine pathway for cysteine biosynthesis (*13*). Complementation of the *S. pombe* mutants with *A. loripes* genes *Acys1a* (Fig. 6A) and *Acys2* (Fig. 6B) rescued the mutant phenotype and enabled the mutant cells to grow in the absence of cysteine in the media. These results confirm that Acys1a is an *O*-succinylserine sulfhydrylase and Acys2 an l-serine-*O*-succinyl transferase, showing that *A. loripes* has the necessary proteins for the biosynthesis of cysteine through the *O*-succinylserine pathway.

## DISCUSSION

It was previously thought that cysteine was exclusively produced through the transsulfuration pathway in animals. Here, we present evidence for the existence of an alternative pathway for cysteine biosynthesis in animals through the analysis of the genome of the complex coral *A. loripes*. We find that the loss of *CBS* in *Acropora* was likely due to the action of transposable elements and identify the existence of an alternative pathway for cysteine biosynthesis through the *O*-succinylserine pathway. This pathway has been recently identified in fungi (*13*) and requires the action of two proteins: Cys2, an l-serine-*O*-succinyltransferase, and Cys1a, an *O*-succinylserine sulfhydrylase. To our surprise, we found the existence of both enzymes not only in the genome of *A. loripes* but also across all animal phyla except for vertebrates, arthropods, and nematodes. Furthermore, these enzymes are found throughout eukaryotes but appear to be absent in higher plants, suggesting that it is an ancestral pathway that was lost independently in plants and several animal lineages. The fact that *A. loripes Acys2* and A*cys1a* can rescue *S. pombe Cys2* and *Cys1a* mutants from cysteine auxotrophy demonstrates that these proteins are indeed functional and likely capable of producing cysteine in vivo in animals.

Our findings highlight that our current understanding of the metabolic capabilities of animals, and life in general, is still incomplete even regarding biosynthetic capabilities that are thought to be well understood. This emphasizes the importance of exercising caution when generalizing and extrapolating findings to emerging systems. In the case of animals, much of our knowledge is derived from studies in a smaller group of model organisms encompassing mammals, arthropods, and nematodes. As shown in this study, this can be misleading and can generate biased conclusions as the characteristics of certain organisms might not necessarily apply to others. Agreeingly, it was previously considered that animals could not synthesize histidine. However, a complete fungal-like histidine biosynthesis pathway was recently identified in robust corals (*33*). Although the functionality of the proteins in this histidine biosynthesis pathway still has to be confirmed, its presence supports the notion that many biosynthetic capabilities are yet to be found.

The reason why the enzymes of the *O*-succinylserine pathway were lost in vertebrates, arthropods, and nematodes is yet to be elucidated. However, differences in domain organization and regulation are found in animal CBSs that could provide an explanation for the repeated loss of this pathway. Mammalian CBS enzymes are known to be more highly regulated than other CBSs. They are positively regulated by the binding of *S*-adenosylmethionine to the CBS domain (*34*), and disruption of this domain results in increased activity and the formation of dimers instead of tetramers (*35*, *36*). Conversely, *Drosophila melanogaster* CBS natively forms dimers and is highly active, and deletion of the C-terminal CBS domains leads to decreased activity (*35*, *36*). In addition, CBS enzymes can have an N-terminal heme binding domain (*37*, *38*), with some vertebrates harboring two (*38*). These domains increase thermal stability (*36*) and activity (*38*, *39*) and have not been found outside metazoans (*37*, *38*, *40*, *41*) (fig. S5). Nematode CBSs, on the other hand, follow a different organization, with longer, highly active monomeric proteins that lack both the N-terminal heme binding and C-terminal CBS domains but have two PLP-dependent domains (*40*). The role of the two PLP-dependent domains is unknown, but it was proposed that the first is involved in protein stability and solubilization, while the second domain is involved in the catalytic activity (*40*). These factors indicate a variable and tight regulation of cysteine biosynthesis, particularly in vertebrates, that is not present in other animals. This required tight regulation may have promoted the conservation of only one pathway for cysteine biosynthesis, yet further investigation is required.

We also identify the existence of a group of proteins greatly resembling Cys1a, composed of photosynthetic algae and oomycetes. Coral dinoflagellate symbionts were found in this clade and were additionally found to have homologs of the Cys2 protein, suggesting that they might also have an alternative pathway for cysteine biosynthesis. Further studies are required to identify whether these proteins can produce cysteine through the *O*-succinylserine pathway or through a similar substrate. The evidence presented in this study shows the biosynthesis of cysteine through an alternative pathway to the transsulfuration pathway in animals. It also shows that corals of the genus *Acropora* have the required machinery for cysteine biosynthesis and thus might not depend on symbiosis with Symbiodiniaceae algae for its acquisition. It is yet to be elucidated whether the loss of cysteine biosynthesis through the transsulfuration pathway in *Acropora* contributes to its increased thermal sensitivity, as evidence for the expression responses of genes involved in cysteine biosynthesis upon thermal stress in *Acropora* and other corals is currently inconclusive (*42*, *43*). It would be interesting to further investigate the contribution of each pathway to cysteine pools in species with both pathways and explore the potential causes favoring the presence of one or two pathways for cysteine biosynthesis in an organism.

## MATERIALS AND METHODS

### DNA extraction, library preparation, and genome size estimation

*A. loripes* samples used for DNA and RNA extraction were collected from Trunk Reef on the Great Barrier Reef (18°35′S, 146°80′E) (fig. S1). High–molecular weight DNA was extracted by grinding frozen sperm in a glass homogenizer filled with homogenization buffer [100 mM tris (pH 9.0), 100 mM EDTA, 100 mM NaCl, and 1% SDS]. After homogenization, proteinase K was added at a concentration of 530 μg/ml, and the homogenate was placed at 70°C for 1 hour. After incubation, 250 μl of KOAc per 1 ml of homogenate was added, mixed thoroughly, incubated for 30 min on ice, and centrifuged at 25,000*g* for 15 min at 4°C. The supernatant was recovered and mixed with 0.8 volumes of isopropanol. The mixture was incubated at room temperature for 15 min and centrifuged at 25,000*g* for 15 min at 20°C to precipitate DNA. The precipitate was washed with 70% ethanol and centrifuged at 25,000*g* for 10 min at 20°C, air-dried, and resuspended overnight at room temperature in 1 ml of resuspension buffer [10 mM tris (pH 7.5) and EDTA 1 mM] with ribonuclease A (100 μg/ml). Genomic DNA was recovered using QIAGEN Genomic-tips following the instructions of the manufacturer and resuspended in resuspension buffer. DNA quality was assessed with NanoDrop (Thermo Fisher Scientific, USA), Qubit 2.0 Fluorometer (Invitrogen, USA), and Fragment Analyzer (Agilent, USA). Two different libraries for genome sequencing were prepared for two different technologies: PacBio and 10x Chromium. The first library was prepared with the SMRTbell Library Preparation Kit (Pacific Biosciences, USA). Fifty-one SMRT cells were run on a PacBio RS II (Pacific Biosciences, USA) system at the Bioscience Core Labs [King Abdullah University of Science and Technology (KAUST), Saudi Arabia], generating 3.2 million reads with an average length of 10.7 kb, resulting in an 85 × coverage. The second library was prepared from 40-kb DNA molecules with the Chromium Genome Library Kit v2 (10x Genomics, USA). The library was sequenced on two lanes of Illumina HiSeq 4000 (Illumina, USA) run in 151–base pair (bp) paired-end mode at the Bioscience Core Labs (KAUST, Saudi Arabia), resulting in 609 million reads and a 210 × coverage.

Genome size and heterozygosity were estimated with GenomeScope (*44*) by measuring abundances of *k*-mers within a set of DNA sequencing reads. For this analysis, *k*-mer frequencies from 10x Chromium DNA sequencing reads stripped of their barcodes were counted with Jellyfish (*45*), and the histogram of *k*-mer abundances was input to GenomeScope (http://qb.cshl.edu/genomescope/) (fig. S3).

### RNA extraction, sequencing, and processing

RNA was extracted from a single nubbin with the Quick-RNA Plant Mini-Prep Kit (R2024) (Zymo Research, USA) following the manufacturer’s instructions. RNA quality was assessed with NanoDrop (Thermo Fisher Scientific, USA), Qubit 2.0 Fluorometer (Invitrogen, USA), and Bioanalyzer (Agilent, USA). RNA sequencing (RNA-seq) libraries were prepared with the TruSeq RNA Library Prep Kit v2 (Illumina, USA) according to the manufacturer’s instructions. RNA-seq libraries were sequenced on one lane of Illumina HiSeq 4000 (Illumina, USA) under 151-bp paired-end mode, resulting in 113 × coverage (based on genome size). Reads were quality trimmed and removed of adapter sequences with Trimmomatic (*46*).

### Genome assembly

SMRT sequencing reads, subsequently referred to as PacBio reads, were error corrected with LoRDEC (*47*) using Chromium 10x linked reads stripped of their barcodes, to resemble Illumina DNA sequencing reads, as reference. Error-corrected PacBio reads were used for genome assembly with Canu (*48*), resulting in a 900-Mb genome. Heterozygosity in the assembly was reduced with Purge Haplotigs (*49*) using parameters -a 41 and -a 66, resulting in two genome versions of 429 (genome_a41) and 541 (genome_a66) Mb, respectively. Assemblies were further corrected and polished with Pilon (*50*) using Minimap2 (*51*) to map error-corrected PacBio reads and BWA-MEM (*52*) to map 10x reads. Genomes were then scaffolded with SSPACE-LongRead (*53*) using PacBio reads, followed by a correction with Pilon, and a further scaffolding with ARCS (*54*), to make use of the barcode information within the 10x reads. Genomes were gap filled with LR_Gapcloser (*55*) and further reduced by removing duplicated or heterozygous regions with HaploMerger2 (*56*). Preliminary annotation of the genomes was performed with BRAKER2 (*18*, *57*, *58*) (for details, see the “Genome annotation and quality assessment” section), and their completeness was assessed with BUSCO (*17*) using the Metazoa v.9 dataset. Missing BUSCO genes from the Metazoa dataset in scaffolds of genome_a41 were searched in genome_a66. Scaffolds from genome_a66 containing missing BUSCO genes in genome_a41 were added to genome_a41. This accounted for only 0.8% of BUSCO genes in the Metazoa dataset in 10 scaffolds comprising 30.9 Mb of genome sequence. From this point in the analysis, only genome_a41 with the added scaffolds was used. To remove duplicated regions after the addition of scaffolds, another round of HaploMerger2 was performed, removing 28.7 Mb of genome sequence, followed by a final step of scaffolding with ARCS, and another round of gap filling with LR_Gapcloser. Genome statistics were computed with stats.sh from BBtools (https://sourceforge.net/projects/bbmap/). A comprehensive explanation and commands used for genome assembly can be found in the Supplementary Materials.

### Genome annotation and quality assessment

The final genome assembly was masked for repetitive elements before annotation by generating a de novo repeat library with RepeatModeler (*59*), which was then used to mask the genome with RepeatMasker and RepeatProteinMask (*60*). To generate hints for genome annotation, RNA-seq data from *A. loripes* was mapped onto the assembled genome with HISAT2 (*61*). Protein prediction on the masked genome was carried out with BRAKER2 using the mapped RNA-seq reads as hints and using the BUSCO-trained Augustus file resulting from running BUSCO on the genome. The completeness of the predicted proteins was assessed by running BUSCO on the predicted proteins with BRAKER2 against the Metazoa v.10 dataset. The quality of the protein prediction was compared against previously sequenced genomes of the genus *Acropora*: an updated *Acropora digitifera* genome (*4*) (GenBank accession GCA_000222465.2), the genome of *Acropora tenuis* (*62*) [Reefgenomics.org (*63*)], and the genome of *Acropora millepora* (*64*) (GenBank accession QTZP00000000). Repetitive elements were also identified for the genomes of *A. digitifera* and *A. tenuis* in the same way as for *A. loripes* (tables S2 to S4). Annotation of the genome of *A. loripes* consisted of performing BLAST (*18*) against three databases: Swiss-Prot, TrEMBL (*27*), and National Center for Biotechnology Information (NCBI) NR (non-redundant) (*65*). Protein domains were identified with InterProScan (*66*), and Gene Ontology terms (*67*) were identified by InterPro entries and by appropriating them from Swiss-Prot–derived annotations. KEGG (*10*) orthologies were retrieved from Swiss-Prot annotations and de novo identified with BlastKOALA (*68*). The complete annotation can be found in data S1.

### Synteny analysis of CBS

The *CBS* gene was searched in the genomes of the following anthozoan taxa: complex corals: *A. digitifera*, *A. loripes*, *A. tenuis*, and *P. lutea* v.1.1 (*69*) (Reefgenomics.org); robust corals: *P. damicornis* v1.0 (*70*) (Reefgenomics.org) and *S. pistillata* v1.0 (*71*) (Reefgenomics.org); Actiniaria: *Actinia equina* v1.0 (*72*) (Reefgenomics.org), *Exaiptasia pallida* (also known as *Exaiptasia diaphana*) (*73*) (GenBank accession GCA_001417965.1) (Reefgenomics.org), and *Nematostella vectensis* v1.0 (*74*) (https://genome.jgi.doe.gov/); and Octocorallia: *Dendronephthya gigantea* (*75*) (GenBank accession GCA_004324835.1). All genomes were reannotated with Swiss-Prot and InterProScan. Loci harboring *CBS* were manually inspected, and neighboring genes were retrieved. To corroborate the presence or absence of synteny, genes showing strong synteny in stony corals had their proteins searched in all other species using TBLASTn against the genomes and BLASTp against their annotations. Preference was given to manual curation over previous annotations, resulting in the inclusion, fusion, or exclusion of genes with respect to their GFF files when sufficient evidence supported it. For the identification of repetitive elements at the *CBS* locus, the RepeatMasker results derived from the de novo repeat libraries generated for all *Acropora* species were analyzed.

### Cysteine biosynthesis pathway analysis

To identify genes that could be involved in the biosynthesis of cysteine in *A. loripes*, its gene annotation was searched for terms related to cysteine. In addition, a BLASTp search was performed with proteins involved in known cysteine biosynthesis pathways against the predicted proteins of *A. loripes*. Genome annotations were also mapped into KEGG pathways (*10*) and analyzed. One protein was identified to have a cysteine synthase 1 domain. Homologs for this putative cysteine synthase were searched in the other anthozoan species. Domains of the putative cysteine synthase and of other cysteine synthases were analyzed with InterProScan (*76*). From these analyses, two proteins resembling *S. pombe* Cys1a and Cys2 were identified in *A. loripes*.

### Phylogenetic analysis

For the classification of *A. loripes* putative Cys1a (Acys1a) and Cys2 (Acys2) proteins, phylogenetic trees were reconstructed. For the phylogenetic reconstruction of cysteine synthases, protein sequences from other cysteine biosynthesis pathways were recovered from individual anthozoan genome projects, Reefgenomics (*63*), NCBI, and UniProt (data S3). For the phylogenetic reconstruction of MetX proteins, only protein sequences from enzymes with validated activities were selected (*13*). For the identification of Cys1a and Cys2 homologs in other species, proteins were recovered through BLASTp in the aforementioned databases. For the identification of phylum-specific sequences, UniProt BLASTp searches were used against the specified phylum. Protein sequences were selected to cover for all possible animal phyla and kingdoms of life (data S3). Phylogenetic reconstructions were carried out with IQ-TREE (*77*) under a maximum likelihood model with 10,000 ultrafast bootstraps (*78*), and trees were visualized with iTOL (*79*).

### Yeast complementation assays

*S. pombe* codon-optimized versions of the *A. loripes Acys1a* and *Acys2* genes were synthesized by Genscript. Genes were cloned into the pREP3xN_ccdB vector carrying the *LEU2* selection marker. The pREP3xN_ccdB vector was a gift from G. Akusjärvi and P. Bjerling (Addgene plasmid #79021; http://n2t.net/addgene:79021; RRID:Addgene_79021). Vectors carrying *A. loripes Acys1a* or *Acys2* or empty were transformed with the lithium acetate method (*80*) into the *S. pombe* haploid strains: wild type (ED666), *ade6-*M210 *ura4*-D18 *leu1*-32; *cys1a*Δ, *ade6*-M216 *ura4*-D18 *leu1*-32 *cys1a*:: *kanMX4*; and *cys2*Δ, *ade6*-M216 *ura4*-D18 *leu1*-32 *cys2*::*kanMX4* (*31*), from Bioneer. Yeast spot assays were carried out in Edinburgh Minimal Media (EMM). In short, *S. pombe* strains were grown in liquid EMM medium with adenine (250 mg/liter) and the corresponding amino acids at a concentration of 250 mg/liter. Strains carrying a construct were grown in the absence of leucine to maintain the plasmid selection. *S. pombe* cells were grown for 2 days at 30°C after which 500 μl was subcultured into 5 ml of liquid EMM. Cultures were allowed to grow for 16 hours and then diluted to an optical density at 600 nm of 0.6 with fresh liquid EMM containing only adenine and without amino acids. For each culture, four 10-fold dilutions were prepared, and 10 μl per sample was spotted in triplicate on EMM plates containing adenine and uracil but with or without the addition cysteine or leucine. Plates were grown for 3 to 5 days before being photographed.

## Supplementary Materials

### This PDF file includes:

Supplementary Materials and Methods Supplementary Text Figs. S1 to S5 Tables S1 to S4 References

## Notes

View/request a protocol for this paper from *Bio-protocol*.
