# Supplementary material for: The coral Acropora loripes genome reveals an alternative pathway for cysteine biosynthesis in animals
Source: Sci Adv. 2022 Sep 23;8(38):eabq0304. doi: 10.1126/sciadv.abq0304 (PMC9506716; doi:10.1126/sciadv.abq0304)
Supplement: Supplementary file 1 — Supplementary Materials and Methods Supplementary Text Figs. S1 to S5 Tables S1 to S4 References [file sciadv.abq0304_sm.pdf]

Science Advances
AAAS

Supplementary Materials for

The coral *Acropora loripes* genome reveals an alternative pathway for cysteine biosynthesis in animals

Octavio R. Salazar *et al.*

Corresponding author: Octavio R. Salazar, octavio.salazarmoya@kaust.edu.sa;
Manuel Aranda, manuel.aranda@kaust.edu.sa

Sci. Adv. **8**, eabq0304 (2022)
DOI: 10.1126/sciadv.abq0304

The PDF file includes:

Supplementary Materials and Methods
Supplementary Text
Figs. S1 to S5
Tables S1 to S4
Legends for data S1 to S3
References

Other Supplementary Material for this manuscript includes the following:

Data S1 to S3

Supplementary Materials and Methods

Acropora loripes genome assembly

A comprehensive explanation and commands used for genome assembly can be found below and the methodology is represented in fig. S2.

Step 1: Error correction of SMRT sequencing PacBio reads using Chromium 10x linked-reads

Since PacBio reads have higher error rates, PacBio reads were corrected using reads coming from 10x Chromium stripped from the barcodes, to resemble Illumina DNA sequencing reads. To correct the reads, LoRDEC (*47*) was run as following:

```
lordec-correct -2 barcodedless_R1.fq,barcodedless_R2.fq -k 21 \
-s3 -T 64 -i All_pacbio.fasta.gz -o \
Acropora_Pacbio_Lorderc_corrected.fasta
```

Step 2: Canu assembly using error corrected PacBio reads

Error corrected PacBio reads were assembled with the Canu (*48*) assembler as follows:

```
canu -p Acropora_loripes -d Acropora genomeSize=540m \
-pacbio-raw \
/path_to_reads/Acropora_Pacbio_Lorderc_corrected.fasta \
"gridOptions=--partition=batch --time=7-00:00:00"
```

The size of this canu generated genome assembly was of 929 Mb. Literature reports geonme sizes for *Acropora spp.* based on C-values to be around 400 Mb.

Step 3: Genome size estimation

Aside of literature information, the genome size of *Acropora loripes* was also checked by k-mer analysis with GenomeScope (*44*) (fig. S3). For this, kmers should be counter with Jellyfish on the barcoded removed 10x reads:

```
jellyfish count -C -m 31 -s 1G -t 64 *.fastq -o reads.jf
jellyfish histo -t 64 reads.jf >read.histo
```

Step 4: Reduction of heterozygosity with Purge Haplotigs

To reduce the heterozigosity, Purge Haplotigs (*49*) was used as follows:

First, PacBio reads are mapped to the genome using Minimap2 (*51*)

```
minimap2 -t 32 -ax map-pb Acropora_loripes.contigs.fasta \
Lordec_trimmed_Canu_Pacbioreads.fasta | samtools view -hF 256 -\
| samtools sort -@ 8 -m 1G -o PSP41_Lordec_sorted.bam -T tmp.ali
```

Then run the purge haplotigs pipeline

```
purge_haplotigs readhist -t 20 -b Canu_Lordec_sorted.bam -g \
Acropora.contigs.fasta
purge_haplotigs contigcov -i Canu_Lordec_sorted.bam.gencov \
-l 4 -m 60 -h 195
```

#In here -a was tested for values ranging from 30-90

```
purge_haplotigs purge -g Acropora.contigs.fasta \
-c coverage_stats.csv -d -b Canu_Lordec_sorted.bam -t 20 -a 70
```

#Genomes were assessed with BUSCO (*17*) and only those maintaining at least the same BUSCO completeness as the original Canu assembly but reducing the overall genome size were

maintained. In this case -a 41 and -a66 produced the best results in terms of size and BUSCO completeness.

```
run_BUSCO.py -i curated.fasta -o busco -m geno -c 24 -l \
/path_to_BUSCO_Dataset/metazoa_odb9
```

From here and forward two genome versions were processed with the same methodology, that produced with the parameter -a 41 and -a 66 producing genomes of 429 Mb and 541 Mb respectively. -a 41 will be used as the main assembly and -a 66 will be used as a complement to later on add scaffolds which are missing from the genome generated with -a 41.

Step 5: Assembly correction and gap closing

PacBio sequencing is prone to indels, to correct this, Illumina reads can be used to fix assembled regions with the program Pilon.

Illumina reads are mapped to the genome using BWA-MEM (*52*)

```
bwa index Canu_La41.fasta
bwa mem -t 32 Canu_La41.fasta barcodedless_R1.fq \
barcodedless_R2.fq | samtools sort -@ 8 -m 1G -o \
Canu_La41_Illumina_sorted.bam -T tmp.ali
```

PacBio reads are mapped to the genome using Minimap2

```
minimap2 -t 32 -ax map-pb Canu_La41.fasta \
Lordec_trimmed_Canu_Pacbioreads.fasta | samtools view -bS - | \
samtools sort -@ 8 -m 1G -o Canu_La41_minimap2_sorted.bam -T \
tmp.ali
```

Then run Pilon

```
java -Xmx400G -jar pilon-1.23.jar --genome Canu_La41.fasta \
```

```
–frags Canu_La41_Illumina_sorted.bam --pacbio \
Canu_La41_minimap2_sorted.bam --output Acropora_pilon 41 \
–outdir Pilon_files --threads 64
```

Step 6: Scaffolding with PacBio reads

The pilon corrected assembly was scaffolded using SSPACE-LongRead (*53*)

```
perl SSPACE-LongRead.pl -c Acropora_pilon41.fasta -p \
Lordec_trimmed_Canu/Lordec_tri mmed_Canu_Pacbioreads.fasta -t 64
```

Step 7: Second round of assembly correction and gap closing

As PacBio data was used to scaffold, Pilon was used to correct any added sequence

```
bwa index scaffolds.fasta
```

Can also use Longranger output to map (as barcodes are in the header of the fastq read)

```
bwa mem -t 32 scaffolds.fasta -p \
/Longranger/LRanger1/outs/interleved_unzip/barco ded.fastq | \
sort -@ 8 -m 1G -o Original_PSP_Illumina_sorted.bam -T tmp.ali
minimap2 -t 32 -ax map-pb scaffolds.fasta \
Lordec_trimmed_Canu_Pacbioreads.fasta | samtools view  -bS - | \
samtools sort -@ 8 -m 1G -o Original_PSP_minimap2_sorted.bam \
-T tmp.ali
java -Xmx400G -jar pilon-1.23.jar --genome scaffolds.fasta \
--frags Original_PSP_Illumina_sorted.bam --pacbio \
Original_PSP_minimap2_sorted.bam --output Acropora_pilon41PSP \
--outdir Pilon_files --threads 64
```

Step 8: Scaffolding with 10X reads

Making use of the barcoded in the 10X reads, the SSPACE-long scaffolded assembly was further scaffolded using ARCS (*54*).

First, 10X reads must be processed with LongRanger

```
longranger basic --fastqs /path_to_10X_reads --id LRanger1
```

#Note, some header can give rise to errors with some programs, changing the fasta headers to simple names might be needed for correct processing.

The LongRanger processed reads are then mapped to the genome with bwa.

```
bwa index Changed_header_Acropora_pilon41PSP.fasta

bwa mem -t 32 Changed_header_Acropora_pilon41PSP.fasta -C -p \

/LongRanger/barcoded.fastq.gz | samtools sort -n -o \

Acro_PSP41_10x_sorted.bam -

arcs --file Changed_header_Acropora_pilon41PSP.fasta -a \

bamfof.txt -b ARCS_AcroPSP41

makeTSVfile.py ARCS_AcroPSP41_original.gv \

ARCS_AcroPSP41_original.tigpair_checkpoint.tsv \

Changed_header_Acropora_pilon41PSP.fasta

touch empty.fof

LINKS -f Changed_header_Acropora_pilon41PSP.fasta -s empty.fof \

-b ARCS_AcroPSP41_original
```

Step 9: Gapclosing with PacBio reads

The scaffolded genome was gapclosed with PacBio reads using LR_Gapcloser (*55*)

```
LR_Gapcloser.sh -i Fixed_header_ARCS_Acropora_Loripes.fasta -l \
Lordec_trimmed_Canu_Pacbioreads.fasta -t 32 -r 5
```

LR_Gapcloser does several iterations of gapclosing, in this case iteration #4 was the last iteration that showed any gap closure improvement.

Step 10: Reduction of duplication

Due to the high level of heterozigosity, duplicated scaffolds were collapsed and removed with HaploMerger2 (*56*)

```
./hm.batchB1.initiation_and_all_lastz gapclosed
./hm.batchB2.chainNet_and_netToMaf gapclosed
./hm.batchB3.haplomerger gapclosed
./hm.batchB4.refine_unpaired_sequences gapclosed
./hm.batchB5.merge_paired_and_unpaired_sequences gapclosed
```

Step 11: Preliminary annotation

The genome was masked by creating a new repeat library with RepeatModeler and masking the genome with RepeatMasker (*60, 81, 82*). RNASeq reads were mapped to the genome with HiSat2(*61*) and were used as hints provided to BRAKER2 (*18, 57, 58, 83, 84*) to perform gene prediction. Genome completeness was assessed with BUSCO.

Masking

```
BuildDatabase -name Acropora41PSP_haplo -engine ncbi \
Fixed_header_gapclosed_ref.fa
RepeatModeler -engine ncbi -pa 32 -database \
Acropora41PSP_haplo > run.out
```

```
RepeatMasker -lib Acropora41PSP_haplo.consensi.fasta -pa 20 \
-xsmall -dir . Fixed_header_gapclosed_ref.fa
RepeatProteinMask -noLowSimple -pvalue 0.0001 -engine ncbi \
Fixed_header_gapclosed_ref.fa.masked
```

Mapping RNASeq reads to create hints

```
hisat2-build Fixed_header_gapclosed_ref.fa Acropora
hisat2 -x Acropora -p 32 -1 \
paired_success_M_17_4547_AL_AD020_L006_R1_001.fastq.normalized_\
K25_C50_pctSD200.fq -2 \
paired_success_M_17_4547_AL_AD020_L006_R2_001.fastq.normalized_\
K25_C50_pctSD200.fq | samtools view -bS - | samtools sort -o \
Hisat_50x_accepted_sorted.bam
```

Gene prediction with BRAKER2

```
braker.pl –genome=Fixed_header_gapclosed_ref.fa.masked.masked \
--bam=Hisat_50x_accepted_sorted.bam -- \
AUGUSTUS_CONFIG_PATH=/path/Augustus-3.3.2/config/ -- \
AUGUSTUS_BIN_PATH=/path/Augustus-3.3.2/bin/ -- \
AUGUSTUS_SCRIPTS_PATH=/path/Augustus-3.3.2/scripts/ \
--cores=32 --softmasking --useexisting \
--species=Aloripes_haplomer41_2_10_rounds –rounds=10 --gff3
```

BUSCO assessment

```
run_BUSCO.py -i augustus.hints.aa -o busco -m proteins -c 20 \
-l /path/BUSCO/metazoa_odb9
```

Step 12: Addition of missing scaffolds

At step #4 was mentioned that 2 different assemblies were processed, one running Purge Haplotigs with parameter -a 41 and another with -a 66. The assembly generated with -a 41 showed a size of the expected genome size whilst -a 66 showed the best BUSCO completeness score, alas with a larger genome size. Both processed genomes were annotated with BRAKER2 and assessed with BUSCO. Scaffolds containing missing BUSCOs in the -a 41 genome but present in the -a 66 genome were added to the -a 41 genome. The difference in missing BUSCOs between both assemblies was of 0.8 %., thus not many scaffolds were added.

Step 13: Reduction of duplication after addition of scaffolds

As extra scaffolds from one assembly were added into another, potential regions of these new scaffolds could be duplications of previously contained scaffolds. For this reason, another round of HaploMerger2 was done.

```
./hm.batchB1.initiation_and_all_lastz \
Haplo41_with_addedPSP66Haplomerge
./hm.batchB2.chainNet_and_netToMaf \
Haplo41_with_addedPSP66Haplomerge
./hm.batchB3.haplomerger Haplo41_with_addedPSP66Haplomerge
./hm.batchB4.refine_unpaired_sequences \
Haplo41_with_addedPSP66Haplomerge
./hm.batchB5.merge_paired_and_unpaired_sequences \
Haplo41_with_addedPSP66Haplomerge
```

Step 14: Final scaffolding with 10X reads

Making use of the barcoded in the 10X reads, the HaploMerger2 reduced assembly was further scaffolded using ARCS.

#Note, some header can give rise to errors with some programs, changing the fasta headers to simple names might be needed for correct processing.

The LongRanger processed reads are then mapped to the genome with bwa.

```
bwa index Changed_header_Haplo41_with_addedPSP66Haplomerge.fasta
bwa mem -t 32 \
Changed_header_Haplo41_with_addedPSP66Haplomerge.fasta \
-C -p /LongRanger/barcoded.fastq.gz | samtools sort -n -o \
Acro_PSP41_10x_sorted.bam -
arcs --file
Changed_header_Haplo41_with_addedPSP66Haplomerge.fasta \
-a bamfof.txt -b ARCS_AcroPSP41
makeTSVfile.py ARCS_AcroPSP41_original.gv \
ARCS_AcroPSP41_original.tigpair_checkpoint.tsv \
Changed_header_Haplo41_with_addedPSP66Haplomerge.fasta
touch empty.fof
LINKS -f \
Changed_header_Haplo41_with_addedPSP66Haplomerge.fasta -s \
empty.fof -b ARCS_AcroPSP41_original
```

Step 15: Gapclosing with PacBio reads

The scaffolded genome was gapclosed with PacBio reads using LR_Gapcloser

```
LR_Gapcloser.sh -i Final_assembly_Acropora_loripes.fasta -l \
Lordec_trimmed_Canu_Pacbioreads.fasta -t 32 -r 5
```

Step 16: Annotation of the final assembly

The final assembly was masked with RepeatMasker and RNASeq reads were mapped to create hints to be given to BRAKER2 for gene prediction.

Masking

```
RepeatMasker -lib Acropora41PSP_haplo.consensi.fasta -pa 20 \
-xsmall -dir . Final_assembly_Acropora_loripes.fasta
RepeatProteinMask -noLowSimple -pvalue 0.0001 -engine ncbi \
Final_assembly_Acropora_loripes.fasta.masked
```

Mapping RNASeq reads

```
hisat2-build Final_assembly_Acropora_loripes.fasta Acropora
hisat2 -x Acropora -p 32 -1 \
paired_success_M_17_4547_AL_AD020_L006_R1_001.fastq.normalized_\
K25_C50_pctSD200.fq -2 paired_success_M_17_4547_AL_AD \
020_L006_R2_001.fastq.normalized_K25_C50_pctSD200.fq | \
samtools view -bS - | samtools sort -o
Hisat_50x_accepted_sorted.bam
```

Gene prediction with BRAKER2:

```
braker.pl \
–genome=Final_assembly_Acropora_loripes.fasta.masked.masked  \
--bam=Hisat_50x_accepted_sorted.bam \
--AUGUSTUS_CONFIG_PATH=/path/Augustus-3.3.2/config/ \
```

```
--AUGUSTUS_BIN_PATH=/path/Augustus-3.3.2/bin/ \
--AUGUSTUS_SCRIPTS_PATH=/path/Augustus-3.3.2/scripts/ \
--cores=32 --softmasking –-useexisting \
--species=Aloripes_Final –-rounds=10 –-gff3
```

BUSCO assessment

```
busco -i augustus.hints.aa -o buscov5 -m proteins -c 20 \
-l metazoa
```

Identification of orthologous groups and phylogenetic analysis

Orthologous groups across anthozoans were identified with OrthoMCL (*85*) by aligning the predicted proteins of the genomes of some of the best annotated anthozoans and *Acropora* species (table S5) against each other with BLASTp (*18, 86*). For this analysis, we included the following symbiotic and non-symbiotic anthozoan taxa: Complex corals: *Acropora digitifera* (*4*), *Acropora loripes*, *Acropora millepora* (*64*), *Acropora tenuis (62)*, and *Porites lutea* v.1.1 (*69*) (Reefgenomics.org); Robust corals: *Pocillopora damicornis* v1.0 (*70*) (Reefgenomics.org) and *Stylophora pistillata* v1.0 (*71*); Actiniaria: *Actinia equina* v1.0 (*72*) (Reefgenomics.org), *Exaiptasia pallida* (also known as *Exaiptasia diaphana*) (*73*) (GenBank accession CA_001417965.1)(Reefgenomics.org), and *Nematostella vectensis* v1.0 (*74*) (https://genome.jgi.doe.gov/); and Octocorallia: *Dendronephthya gigantea* (*75*) (GenBank accession GCA_004324835.1). All of these cnidarians harbor intracellular Symbiodiniaceae, except for *Actinia equina, Nematostella vectensis* and *Dendronephthya gigantea.* All proteins were re-annotated with InterProScan (*66*) and by performing BLAST against the Swiss-Prot database (*27*). GO terms (*67, 87*) for each protein were obtained for both annotation procedures and analyzed separately. Lists of orthogroups belonging to each species were retrieved and

combined to generate lists at an order level to form Complex corals, Robust corals, and Actiniaria. The Symbiotic dataset (Symbiodiniaceae-containing cnidarians) was generated by combining Complex corals, Robust corals, and *Exaiptasia pallida*. Orthogroup intersections were identified with the Venn diagram creator of the University of Gent (http://bioinformatics.psb.ugent.be/webtools/Venn/). GO enrichment analyses on the orthogroup lists were performed with BINGO (*88*, *89*) using a Benjamini and Hochberg false discovery rate correction of 0.05. Enriched terms were analyzed with REVIGO (*90*) to collapse related terms.

Phylogenetic reconstruction was performed by selecting single copy orthogroups present in every species with sequences of at least 200 amino acids. Sequences for 442 orthogroups were recovered from each species and aligned with MUSCLE (*91*). Poorly aligned positions were eliminated, and informative sites were concatenated with Gblocks (*92, 93*) and run with default parameters with the addition of -b5=h. The most appropriate protein evolutionary model was selected with ModelTest-NG (*94*) and the phylogenetic analysis was performed with maximum likelihood inference with RAxML-NG (*95*) under a JTT+I+G4 model and 1000 bootstrap replicates.

Supplementary Text

Genome assembly

Libraries from high molecular weight DNA were prepared for sequencing with two different technologies: SMRT Sequencing from Pacific Biosciences and Chromium from 10x Genomics. A total of 3.2 million SMRT Sequencing reads with a 10.7 kb read length average were generated, resulting in 85x coverage. Similarly, 609 million barcoded Chromium 10x reads derived from 40 kb DNA molecules were generated, comprising 210x coverage. Genome size was estimated with GenomeScope (*44*), resulting in an expected size of ~405 Mb (fig. S3). This is in line with other sequenced *Acropora* species, with genome sizes ranging between 370 - 480 Mb (*4, 64, 69, 96*).

Initial assemblies with Canu (*48*), using PacBio reads, led to a genome assembly of 900 Mb, more than twice the expected size. Assembling with the diploid aware assembler Supernova (*97*) using 10x reads, resulted in a smaller 517 Mb assembly. While the Supernova assembly was closer to the expected genome size, it was extremely fragmented with 47,000 scaffolds. The increased assembly size is likely due to a high degree of heterozygosity in the genome, as the *k-mer* profile by GenomeScope shows a bimodal distribution (fig. S3). Bimodal distributions are characteristic of highly heterozygous genomes, while a simple Poisson distribution is observed in homozygous genomes (*98*). Moreover, GenomeScope predicted a heterozygosity rate of 2.2 % (fig. S4), which is greater than the 1.9% for the highly heterozygous genome of the Pacific oyster *Crassostrea gigas* (*99*). *C. gigas* has been commonly used as a reference for highly heterozygous genomes (*100*) and has been used while benchmarking different bioinformatic tools such as GenomeScope and the repeat aware assembler Platanus (*98*). This resulted in an estimation in

line with literature of ~400 Mb. However, Canu produced an assembly of more than 900 Mb. This was likely due to the high degree of heterozygosity of the extracted DNA.

The high heterozygosity, which makes genome assembly extremely challenging, is likely due to the combined effects of collecting DNA from a wild organism and extracting DNA from the coral sperm, which consists of a mix of non-identical haploid genotypes as the sperm is formed via meiosis. To reduce the negative effects of heterozygosity, we assembled the genome in several steps. Briefly, PacBio reads were error corrected with LoRDEC (*47*) using debarcoded 10x reads. The error corrected reads were then assembled with Canu and the assembly was collapsed, to remove redundant regions, with Purge Haplotigs (*49*) and HaploMerger2 (*56*). The assembly was then scaffolded using 10x barcoded reads with ARCS (*54*). For more details about the complete assembly process please refer to Supplementary Methods.

The final genome assembly is comprised of 335 scaffolds and 398 contigs with a total size of 401 Mb (Table 1), resulting in the least fragmented coral genome recovered to date. The genome has a Contig N50 of 2.35 Mb and a Scaffold N50 of 2.82 Mb, with 0.002 % of unknown bases (Ns) (table S1). The GC content is 38.99 %, in line with other *Acropora* species which range from 38.85 – 39.07 % (*4, 64, 69*). A comparison of the assembly with other sequenced *Acropora* species can be found in Table 1.

Repetitive elements in *Acropora*

Repetitive element contents were compared across *Acropora* by generating *de novo* repeat libraries for the genomes of *Acropora loripes, Acropora tenuis (62),* and *Acropora digitifera (4),* with RepeatModeler followed by the masking of their genomes with RepeatMasker. Repetitive elements accounted for 43.13 % of the *A. loripes* genome (table S1), similar to the 43.39 %

found in *A. tenuis* (table S2), but higher than the 30.99 % and 34.55 % of *A. digitifera* (table S3) and *A. millepora* (*64*), respectively (Table 1). In all genomes, interspersed repeats accounted for the majority of the repetitive sequences. Of the classified repeats, Long Interspersed Nuclear Elements (LINEs) were the most abundant repetitive element, followed by DNA transposable elements (tables S1-3).

Gene orthology with other Anthozoans

The genome annotation of *A. loripes* was compared, by means of orthology, against some of the best annotated anthozoan genomes (table S4). Groups of orthologous proteins were identified with OrthoMCL (*85*) across the genomes of the taxonomic group of Complex corals: *Acropora digitifera*, *Acropora loripes*, *Acropora millepora*, *Acropora tenuis*, and *Porites lutea* (*69*); the taxonomic group of Robust corals: *Pocillopora damicornis* (*70*) and *Stylophora pistillata* (*71*); the order Actiniaria: *Actinia equina* (*72*), *Exaiptasia pallida* (also known as *Exaiptasia diaphana*) (*73*), and *Nemtastella vectensis* (*74*); and the anthozoan subclass Octocorallia: *Dendronephthya gigantea* (*75*). A total of 32,207 orthogroups were identified comprising 280,719 proteins (data S2). From the 32,207 orthogroups, 5,411 were shared across all species. Of these, 710 consisted of single copy genes, of which 422 were used to reconstruct a phylogenetic tree using the octocoral *D. gigantea*, as an outgroup (fig. S4). The phylogenetic tree clearly resolved Actiniaria, Complex corals, and Robust corals, showing the calcifying Scleractinia (Complex and Robust corals) diverging from the non-calcifying Actiniaria.

When comparing *Acropora* species, 10,230 orthogroups were shared across all species (fig. S4), of which 312 were unique to the genus *Acropora*. Gene Ontology (GO) enrichment analyses of the unique orthogroups in *Acropora*, revealed enrichment in terms related to the

regulation of apoptosis (data S2). Interestingly, this enrichment was also found in orthogroups unique to Complex (5,557) and Robust corals (1,534) (fig. S4 and data S2); however, this was not observed in orthogroups unique to the Complex coral *P. lutea* (data S2), suggesting that *P. lutea* may have lost some of the genes involved in the regulation of apoptosis. Unique orthogroups of Complex corals also revealed an enrichment in terms related to immune response and G-coupled receptor signaling pathway (data S2). Moreover, it also revealed an enrichment of the term modification of morphology or physiology of other organism involved in symbiotic interaction. This is interesting as stony corals form symbiosis with dinoflagellates, although the term was not enriched in Robust corals. When examining orthogroups unique and present in all species forming symbiosis with Symbiodiniaceae, i.e. Complex, Robust corals and *Exaiptasia pallida*, only 37 orthogroups were identified. Of these, the term BRCA1-A complex was enriched, which is involved in DNA damage repair (*101*). However, the number of orthogroups unique and present in all symbiotic species was too small for a robust statistical analysis of GO enrichment and the remaining orthogroups should not be dismissed. Noteworthy, most of these orthogroups had terms related to transmembrane transporter activities, primarily ion channels. Ion channels are associated with microbial symbiosis in plants (*102*) and it is possible they also play a role in cnidarian-Symbiodiniaceae symbiosis. Similar to Complex corals, unique orthogroups in Actiniaria (5,436) were also enriched in G-coupled receptor signaling pathway but not in genes related to immune response or symbiosis. The unique orthogroups of the octocoral *D. gigantea* (1,174) were enriched in terms related to uridylyltransferase activity, which is involved in the regulation of nitrogen assimilation (*103, 104*), and positive regulation of developmental process; both terms related to growth. Results from the GO enrichment analyses can be found in data S2.

O-acetylserine sulfhydrylases in nematodes

Interestingly, O-acetylserine sulfhydrylases (CysK) resembling those in plants have been identified in *C. elegans* (cysl proteins), where *in vitro* assays showed that these proteins can produce cysteine (*105*). This would suggest for an alternative cysteine biosynthesis through the sulfate assimilation pathway that is only found in nematodes and missing in all other animals. However, the remaining required proteins for the biosynthesis of cysteine through the sulfate assimilation pathway are not found in *C. elegans* and the biosynthesis of cysteine is considered to be carried out through the transsulfuration pathway (*105*). Furthermore, these enzymes are not consistently found across nematodes (*105*) and have been associated with other functions such as response to hypoxia, hydrogen sulfide, and hydrogen cyanide (*105-107*). This contrasts with our findings, in which both enzymes required for cysteine biosynthesis through the O-succinylserine pathway are present and conserved across animal lineages, supporting the role of a conserved function.

Supplementary Figures

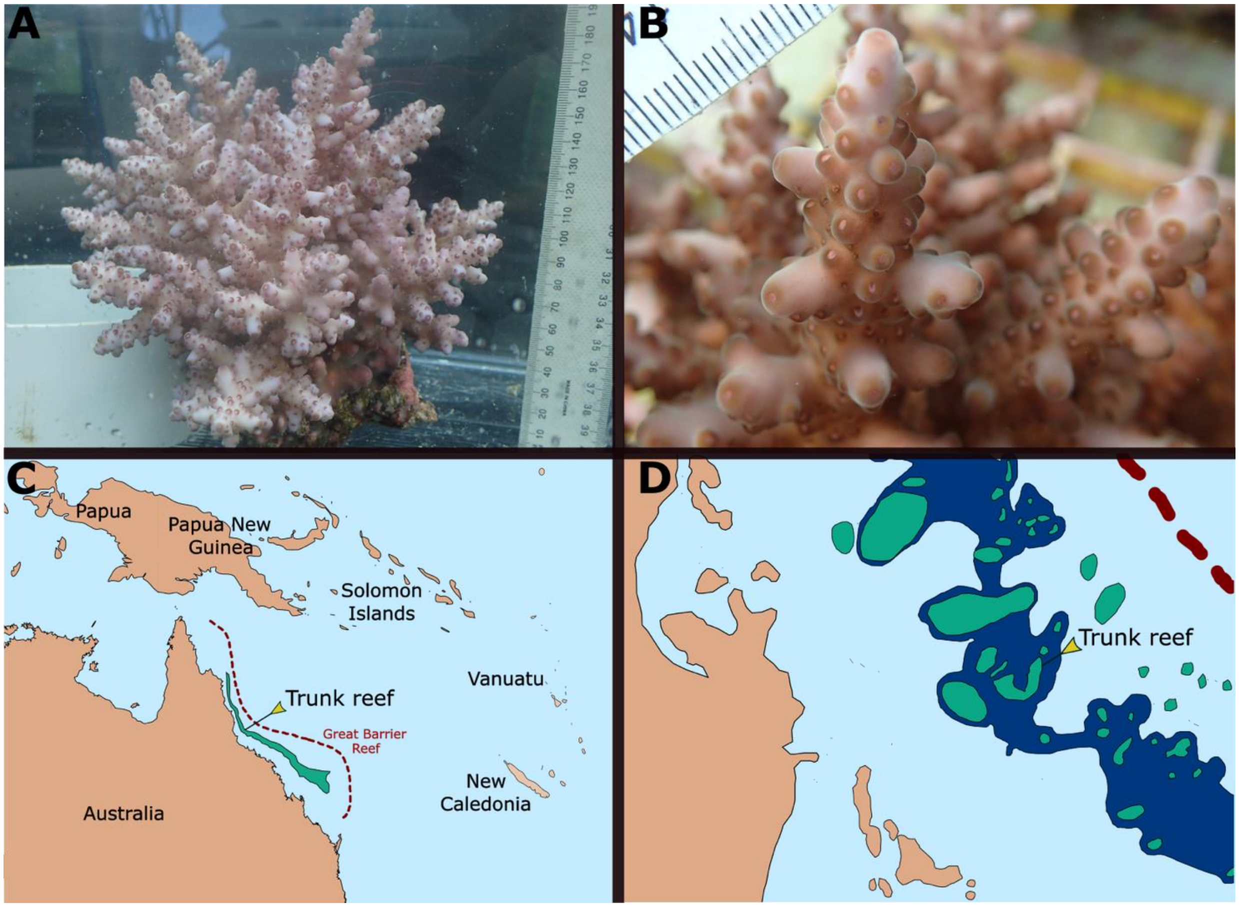

Figure S1. *Acropora loripes* sample collection. DNA was isolated from samples collected at coordinates 18°35′S, 146°80′E, the Trunk reef in the Great Barrier Reef. (**A**), *Acropora loripes* colony. (**B**), *Acropora loripes* polyps. (**C**), Localization of the Trunk reef within the Great Barrier Reef. (**D**), Zoomed-in Trunk reef area.

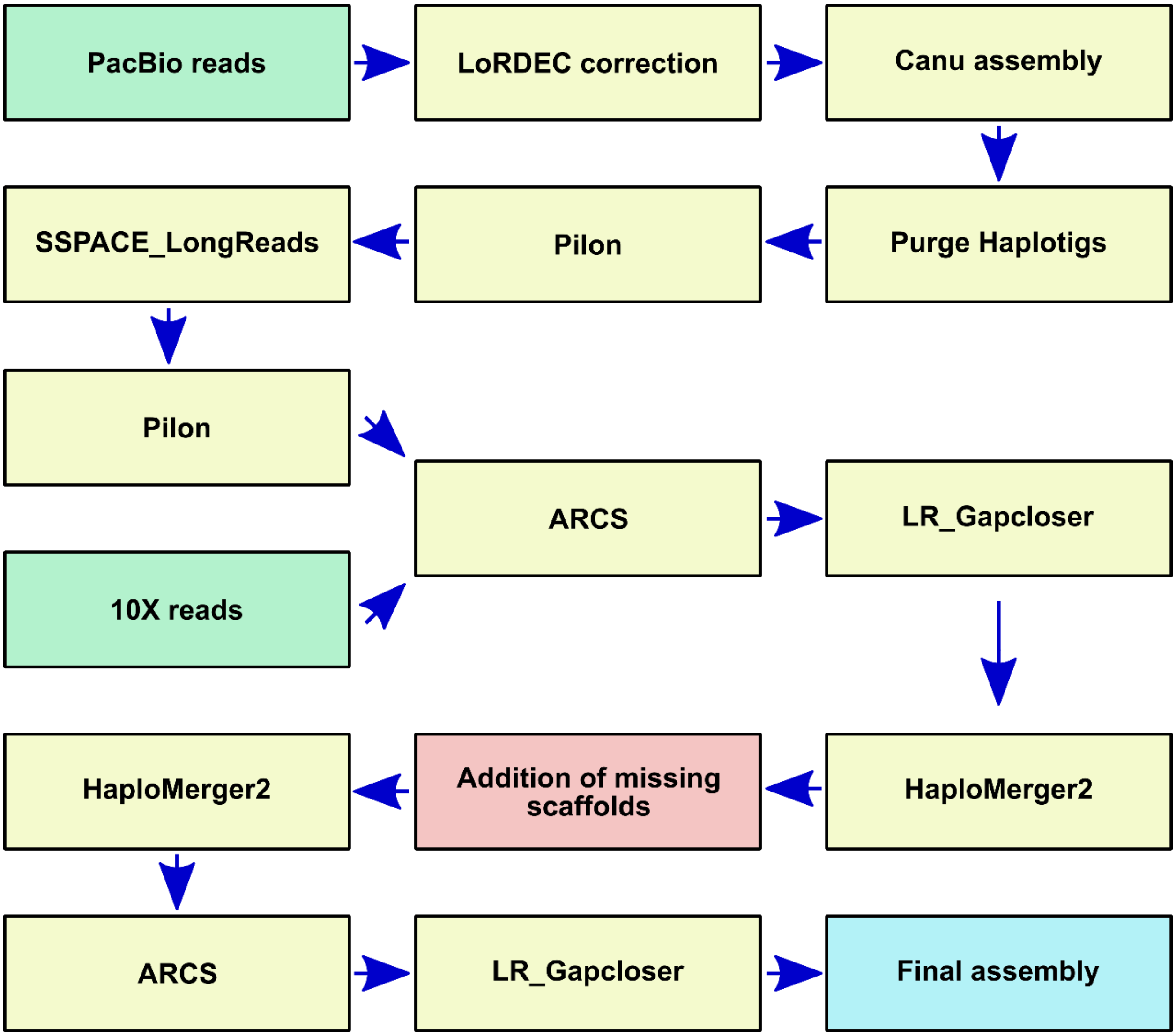

Figure S2. Overview of the methodology for the genome assembly of *A. loripes*.

A

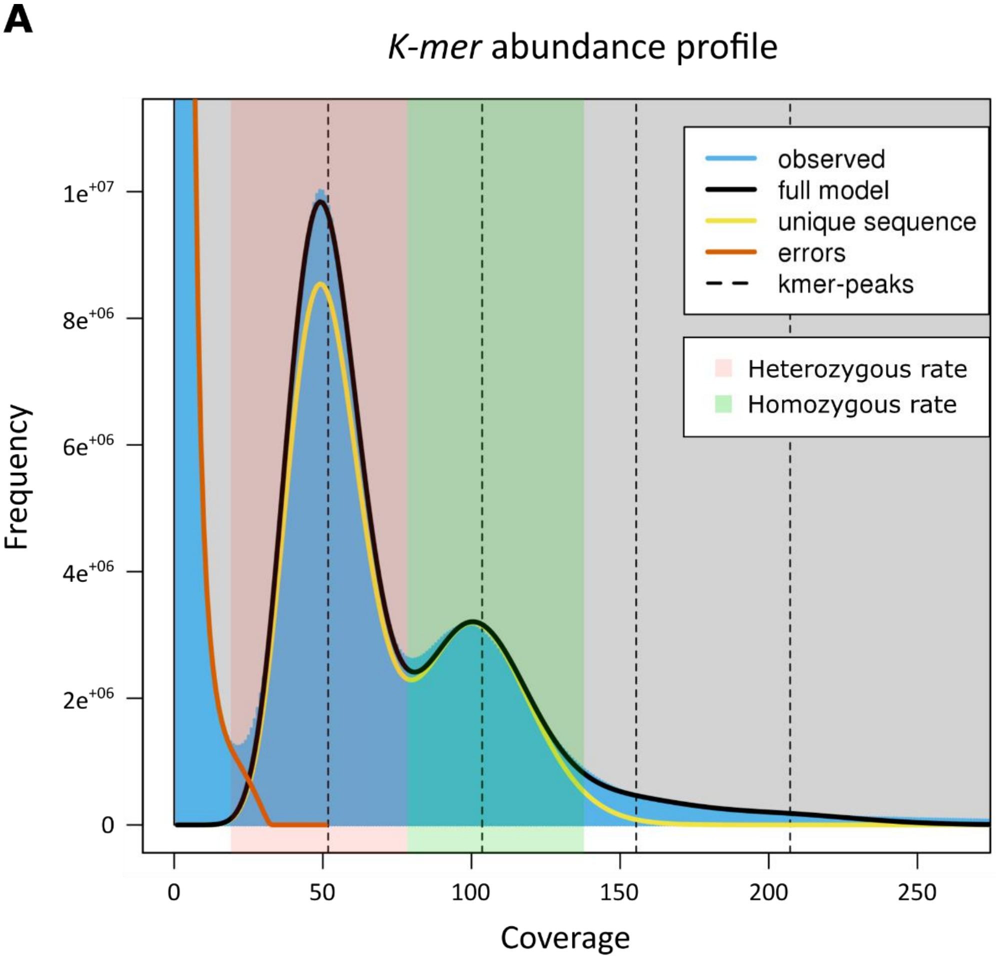

B

Property	Minimum	Maximum
Genome Haploid Length (Mb)	405.50	406.15
Heterozygosity (%)	2.23	2.25
Model Fit (%)	94.07	96.86
Read Error Rate (%)	0.87	0.87

Figure S3. Genome size and heterozygosity estimation of *Acropora loripes* by GenomeScope. (**A**), *K-mer* abundance profile plot showing a bimodal distribution. Shaded in red, the peak close to 50x coverage represents the heterozygosity rate. Shaded in green, the peak near 100x coverage represents the homozygosity rate. (**B**), Minimum and maximum estimated values by GenomeScope.

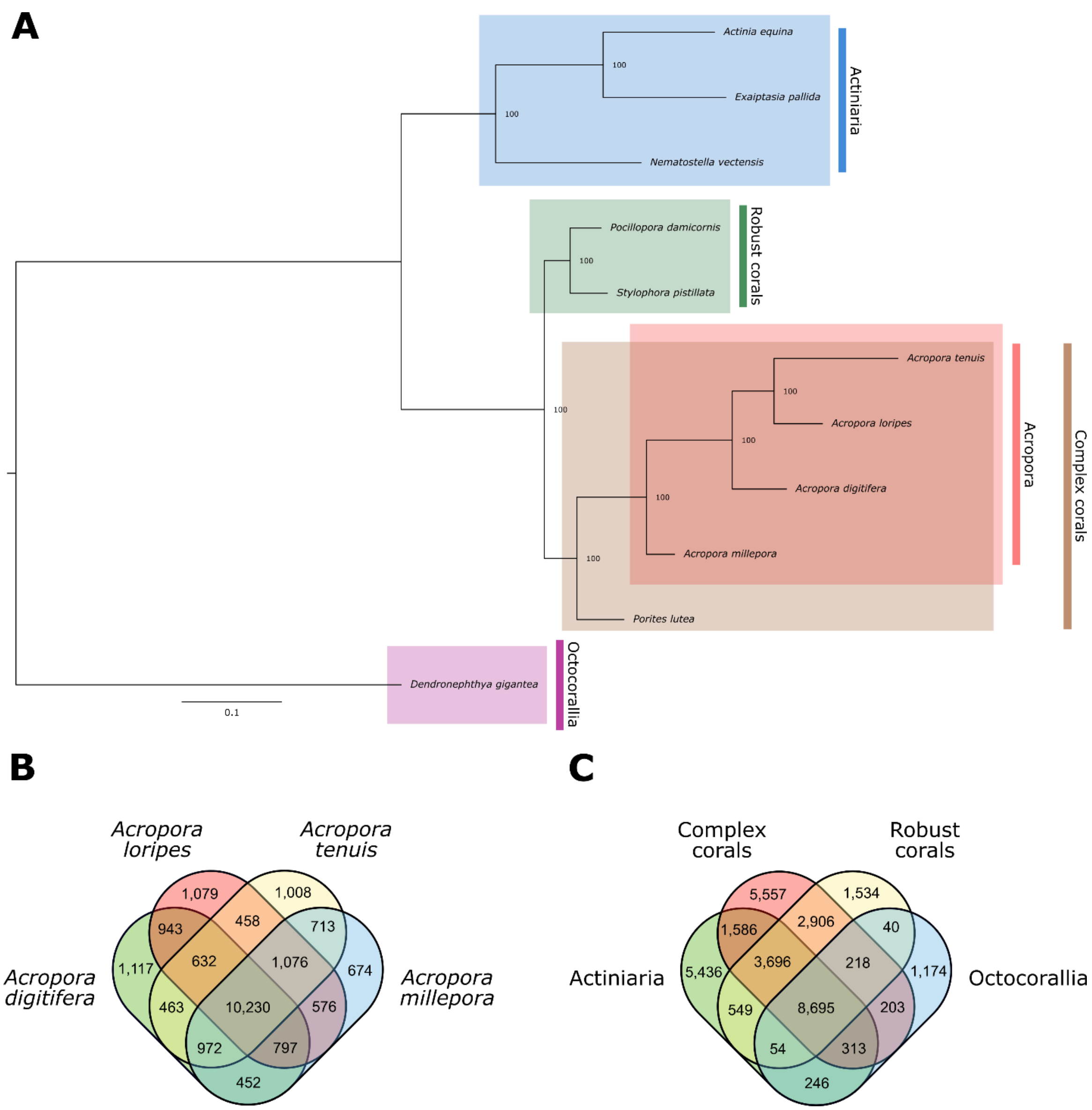

Figure S4. Orthogroup relationships across species. (**A**) Phylogenetic analysis of 422 single copy orthologs of at least 200 amino acids in length. In blue, Actiniaria, in green Robust corals, in red *Acropora*, in brown Complex corals, and in purple Octocorallia as outgroup. Numbers at nodes represent bootstrap values. (**B**) Number of orthogroups shared across the *Acropora* species: *Acropora digitifera*, *Acropora loripes*, *Acropora tenuis*, and *Acropora millepora*. (**C**) Number of orthgroups shared across Actiniaria, Complex corals, Robust corals, and Octocorallia.

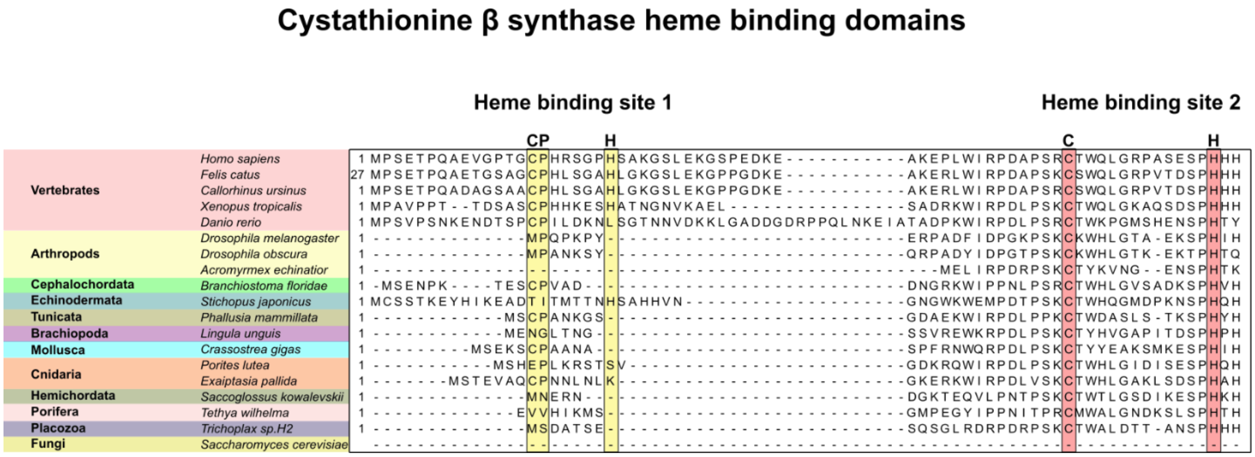

Figure S5. Heme binding domain conservation in cystathionine β synthase proteins.

Alignment of CBS proteins from different animal phyla and fungi. The positions of the two heme binding sites are marked in yellow, and red. Required residues for heme biding are marked as bold letters above the alignment. The first heme binding site corresponds to an intrinsically disordered region only found in some vertebrates. The second heme binding site is conserved across all animals but absent in other eukaryotes. Nematode sequences were not included as they possess a second PLP-dependent domain in this region and lack the heme binding sites.

Supplementary Tables

Table S1. ***Acropora loripes*** **repeat sequences.**

sequences:	335
total length:	401875658 bp (401866882 bp excl N/X-runs)
GC level:	38.99%
bases masked:	173316598 bp (43.13 %)

		Number of elements	length occupied (bp)	percentage of sequence
SINEs:		2827	401066	0.1
	ALUs	0	0	0
	MIRs	1345	175090	0.04
LINEs:		67151	24581111	6.12
	LINE1	1860	1245984	0.31
	LINE2	18667	7208772	1.79
	L3/CR1	1599	568304	0.14
LTR elements:		10138	7725567	1.92
	ERVL	0	0	0
	ERVL-MaLRs	0	0	0
	ERV_classI	0	0	0
	ERV_classII	0	0	0
DNA elements:		23812	10307773	2.56
	hAT-Charlie	0	0	0
	TcMar-Tigger	90	12813	0
Unclassified:		533138	129858485	32.31
Total interspersed repeats:			172874002	43.02
Small RNA:		0	0	0
Satellites:		0	0	0
Simple repeats:		4637	803129	0.2
Low complexity:		0	0	0

* most repeats fragmented by insertions or deletions have been counted as one element

Table S2. ***Acropora tenuis*** **repeat sequences.**

sequences:	614
total length:	486812518 bp (486812518 bp excl N/Xruns)
GC level:	39.07%
bases masked:	211231059 bp (43.39 %)

		Number of elements	length occupied (bp)	percentage of sequence
SINEs:		3395	529668	0.11
	ALUs	0	0	0
	MIRs	3395	529668	0.11
LINEs:		73617	30419090	6.25
	LINE1	2062	1879682	0.39
	LINE2	16976	7368475	1.51
	L3/CR1	2543	914152	0.19
LTR elements:		12173	8783833	1.8
	ERVL	0	0	0
	ERVL-MaLRs	0	0	0
	ERV_classI	0	0	0
	ERV_classII	0	0	0
DNA elements:		29746	13030154	2.68
	hAT-Charlie	0	0	0
	TcMar-Tigger	0	0	0
Unclassified:		625139	151547221	31.13
Total interspersed repeats:			204309966	41.97
Small RNA:		0	0	0
Satellites:		0	0	0
Simple repeats:		101102	6465022	1.33
Low complexity:		12499	629466	0.13

* most repeats fragmented by insertions or deletions have been counted as one element

Table S3. *Acropora digitifera* repeat sequences.

sequences: 2421
total length: 447497157 bp (379294781 bp excl N/X runs)
GC level: 39.04%
bases masked: 138680860 bp (30.99 %)

		Number of elements	length occupied (bp)	percentage of sequence
SINEs:		2595	373530	0.08
	ALUs	0	0	0
	MIRs	2595	373530	0.08
LINEs:		52702	16801111	3.75
	LINE1	217	143927	0.03
	LINE2	14912	4984442	1.11
	L3/CR1	1367	532175	0.12
LTR elements:		9351	4917141	1.1
	ERVL	0	0	0
	ERVL-MaLRs	0	0	0
	ERV_classI	0	0	0
	ERV_classII	0	0	0
DNA elements:		14900	5078729	1.13
	hAT-Charlie	0	0	0
	TcMar-Tigger	0	0	0
Unclassified:		531913	106909439	23.89
Total interspersed repeats:			134079950	29.96
Small RNA:		0	0	0
Satellites:		0	0	0
Simple repeats:		68010	4233139	0.95
Low complexity:		9717	458620	0.1

* most repeats fragmented by insertions or deletions have been counted as one element

Table S4. BUSCO scores for protein predictions of anthozoan species used for orthology analysis against the Metazoa dataset V10 (954 proteins)

Species	Complete	Complete Single copy	Complete Duplicated	Fragmented	Missing
Acropora digitifera	77.5	63.5	14	10.2	12.3
Acropora loripes	96.1	89.5	6.6	1.7	2.2
Acropora millepora	88	87.3	0.7	6.5	5.5
Acropora tenuis	88.6	87.7	0.9	4.8	6.6
Actinia equina	95.6	53	42.6	2.6	1.8
Dendronephthya gigantea	95.2	73.5	21.7	0.8	4
Exaiptasia pallida	85.3	82.3	3	6.4	8.3
Nematostella vectensis	92.9	90.8	2.1	4.3	2.8
Pocillopora damicornis	87.9	87.7	0.2	5.7	6.4
Porites lutea	91.7	89.6	2.1	4.1	4.2
Stylophora pistillata	87.3	86.9	0.4	5.2	7.5

Data S1. (Supplementary Data File 1)

Contains the files concerning the *Acropora loripes* genome protein prediction and annotation.

Data S2. (Supplementary Data File 2)

Contains gene orthology and GO enrichment analyses based on different anthozoan genomes.

Data S3. (Supplementary Data File 3)

Contains the information and sequences of cysteine synthases and MetX enzymes used in this study.

REFERENCES AND NOTES

1. A. J. L. Cooper, Biochemistry of sulfur-containing amino acids. *Annu. Rev. Biochem.* **52**, 187–222 (1983).

2. S. H. Mudd, J. D. Finkelstein, F. Irreverre, L. Laster, Homocystinuria: An enzymatic defect. *Science* **143**, 1443–1445 (1964).

3. S. H. Mudd, F. Skovby, H.L. Levy, K.D. Pettigrew, B. Wilcken, R.E. Pyeritz, G. Andria, G.H. Boers, I.L. Bromberg, R. Cerone, The natural history of homocystinuria due to cystathionine beta-synthase deficiency. *Am. J. Hum. Genet.* **37**, 1–31 (1985).

4. C. Shinzato, E. Shoguchi, T. Kawashima, M. Hamada, K. Hisata, M. Tanaka, M. Fujie, M. Fujiwara, R. Koyanagi, T. Ikuta, A. Fujiyama, D. J. Miller, N. Satoh, Using the *Acropora* digitifera genome to understand coral responses to environmental change. *Nature* **476**, 320–323 (2011).

5. C. Shinzato, K. Khalturin, J. Inoue, Y. Zayasu, M. Kanda, M. Kawamitsu, Y. Yoshioka, H. Yamashita, G. Suzuki, N. Satoh, Eighteen coral genomes reveal the evolutionary origin of *Acropora* strategies to accommodate environmental changes. *Mol. Biol. Evol.* **38**, 16–30 (2020).

6. V. Schoepf, M. Stat, J. L. Falter, M. T. McCulloch, Limits to the thermal tolerance of corals adapted to a highly fluctuating, naturally extreme temperature environment. *Sci. Rep.* **5**, 17639 (2015).

7. Y. Loya, K. Sakai, K. Yamazato, Y. Nakano, H. Sambali, R. van Woesik, Coral bleaching: The winners and the losers. *Ecol. Lett.* **4**, 122–131 (2001).

8. T. Leustek, M. N. Martin, J. A. Bick, J. P. Davies, Pathways and regulation of sulfur metabolism revealed through molecular and genetic studies. *Annu. Rev. Plant. Physiol. Plant. Mol. Biol.* **51**, 141–165 (2000).

9. E. Guédon, I. Martin-Verstraete, Cysteine Metabolism and Its Regulation in Bacteria, in *Amino Acid Biosynthesis ~ Pathways, Regulation and Metabolic Engineering*, V. F. Wendisch, Ed. (Springer Berlin Heidelberg, 2006), pp. 195–218.

10. M. Kanehisa, S. Goto, KEGG: Kyoto Encyclopedia of Genes and Genomes. *Nucleic Acids Res.* **28**, 27–30 (2000).

11. M. Kanehisa, Toward understanding the origin and evolution of cellular organisms. *Protein Sci.* **28**, 1947–1951 (2019).

12. R. Caspi, R. Billington, L. Ferrer, H. Foerster, C. A. Fulcher, I. M. Keseler, A. Kothari, M. Krummenacker, M. Latendresse, L. A. Mueller, Q. Ong, S. Paley, P. Subhraveti, D. S. Weaver, P. D. Karp, The MetaCyc database of metabolic pathways and enzymes and the BioCyc collection of pathway/genome databases. *Nucleic Acids Res.* **42**, D471–D480 (2016).

13. K. Bastard, A. Perret, A. Mariage, T. Bessonnet, A. Pinet-Turpault, J.L. Petit, E. Darii, P. Bazire, C. Vergne-Vaxelaire, C. Brewee, A. Debard, V. Pellouin, M. Besnard-Gonnet, F. Artiguenave, C. Médigue, D. Vallenet, A. Danchin, A. Zaparucha, J. Weissenbach, M. Salanoubat, V. de Berardinis, Parallel evolution of non-homologous isofunctional enzymes in methionine biosynthesis. *Nat. Chem. Biol.* **13**, 858–866 (2017).

14. M. Grynberg, J. Topczewski, A. Godzik, A. Paszewski, The *Aspergillus nidulans cysA* gene encodes a novel type of serine *O*-acetyltransferase which is homologous to homoserine *O*-acetyltransferases.. *Microbiology (Reading)* **146**, 2695–2703 (2000).

15. Y. Ma, R. Sugiura, M. Saito, A. Koike, S. O. Sio, Y. Fujita, K. Takegawa, T. Kuno, Six new amino acid-auxotrophic markers for targeted gene integration and disruption in fission yeast. *Curr. Genet.* **52**, 97–105 (2007).

16. Y. Fujita, K. Takegawa, Characterization of two genes encoding putative cysteine synthase required for cysteine biosynthesis in *Schizosaccharomyces pombe. Biosci. Biotechnol. Biochem.* **68**, 306–311 (2004).

17. F. A. Simão, R. M. Waterhouse, P. Ioannidis, E. V. Kriventseva, E. M. Zdobnov, BUSCO: Assessing genome assembly and annotation completeness with single-copy orthologs. *Bioinformatics* **31**, 3210–3212 (2015).

18. C. Camacho, G. Coulouris, V. Avagyan, N. Ma, J. Papadopoulos, K. Bealer, T. L. Madden, BLAST^{+}: Architecture and applications. *BMC Bioinform.* **10**, 421 (2009).

19. J. Cappello, S. M. Cohen, H. F. Lodish, *Dictyostelium* transposable element DIRS-1 preferentially inserts into DIRS-1 sequences. *Mol. Cell. Biol.* **4**, 2207–2213 (1984).

20. M. B. Evgen'ev, H. Zelentsova, N. Shostak, M. Kozitsina, V. Barskyi, D.-H. Lankenau, V. G. Corces, *Penelope*, a new family of transposable elements and its possible role in hybrid dysgenesis in *Drosophila virilis*. *Proc. Natl. Acad. Sci. U.S.A.* **94**, 196–201 (1997).

21. E. F. Hoff, H. L. Levin, J. D. Boeke, *Schizosaccharomyces pombe* retrotransposon Tf2 mobilizes primarily through homologous cDNA recombination. *Mol. Cell. Biol.* **18**, 6839–6852 (1998).

22. K. Mino, K. Ishikawa, A novel *O*-phospho-L-serine sulfhydrylation reaction catalyzed by *O*-acetylserine sulfhydrylase from *Aeropyrum pernix* K1. *FEBS Lett.* **551**, 133–138 (2003).

23. E. M. Steiner, D. Böth, P. Lössl, F. Vilaplana, R. Schnell, G. Schneider, CysK2 from *Mycobacterium tuberculosis* is an *O*-phospho-L-serine-dependent *S*-sulfocysteine synthase. *J. Bacteriol.* **196**, 3410–3420 (2014).

24. D. Agren, R. Schnell, W. Oehlmann, M. Singh, G. Schneider, Cysteine synthase (CysM) of *Mycobacterium tuberculosis* is an *O*-phosphoserine sulfhydrylase: Evidence for an alternative cysteine biosynthesis pathway in mycobacteria. *J. Biol. Chem.* **283**, 31567–31574 (2008).

25. S. E. O'Leary, C. T. Jurgenson, S. E. Ealick, T. P. Begley, *O*-phospho-L-serine and the thiocarboxylated sulfur carrier protein CysO-COSH are substrates for CysM, a cysteine synthase from *Mycobacterium tuberculosis*. *Biochemistry* **47**, 11606–11615 (2008).

26. G. D. Westrop, G. Goodall, J. C. Mottram, G. H. Coombs, Cysteine biosynthesis in *Trichomonas vaginalis* involves cysteine synthase utilizing *O*-phosphoserine. *J. Biol. Chem.* **281**, 25062–25075 (2006).

27. UniProt Consortium, UniProt: The universal protein knowledgebase in 2021. *Nucleic Acids Res.* **49**, D480–D489 (2020).

28. M. P. Ferla, W. M. Patrick, Bacterial methionine biosynthesis. *Microbiology (Reading)* **160**, 1571–1584 (2014).

29. B. Maurya, M. Colaço, J. Wouters, L. Pochet, S. Misquith, Key amino acid residues in homoserine-acetyltransferase from *M. tuberculosis* give insight into the evolution of MetX family of enzymes – HAT, SAT and HST. *Biochimie* **189**, 13–25 (2021).

30. K. Oda, Y. Matoba, T. Kumagai, M. Noda, M. Sugiyama, Crystallographic study to determine the substrate specificity of an L-serine-acetylating enzyme found in the D-cycloserine biosynthetic pathway. *J. Bacteriol.* **195**, 1741–1749 (2013).

31. D.-U. Kim, J. Hayles, D. Kim, V. Wood, H.O. Park, M. Won, H.S. Yoo, T. Duhig, M. Nam, G. Palmer, S. Han, L. Jeffery, S.T. Baek, H. Lee, Y. S. Shim, M. Lee, L. Kim, K.S. Heo, E. J. Noh, A.R. Lee, Y.J. Jang, K.S. Chung, S.J. Choi, J.Y. Park, Y. Park, H. M. Kim, S.K. Park, H.J. Park, E.J. Kang, H. B. Kim, H.S. Kang, H.M. Park, K. Kim, K. Song, K. B. Song, P. Nurse, K.L. Hoe, Analysis of a genome-wide set of gene deletions in the fission yeast *Schizosaccharomyces pombe*. *Nat. Biotechnol.* **28**, 617–623 (2010).

32. J. Brzywczy, A. Paszewski, Sulfur amino acid metabolism *in Schizosaccharomyces pombe*: Occurrence of two O-acetylhomoserine sulfhydrylases and the lack of the reverse transfulfuration pathway. *FEMS Microbiol. Lett.* **121**, 171–174 (1994).

33. H. Ying, I. Cooke, S. Sprungala, W. Wang, D. C. Hayward, Y. Tang, G. Huttley, E. E. Ball, S. Forêt, D. J. Miller, Comparative genomics reveals the distinct evolutionary trajectories of the robust and complex coral lineages. *Genome Biol.* **19**, 175 (2018).

34. J. W. Scott, S. A. Hawley, K. A. Green, M. Anis, G. Stewart, G. A. Scullion, D. G. Norman, D. G. Hardie, CBS domains form energy-sensing modules whose binding of adenosine ligands is disrupted by disease mutations. *J. Clin. Invest.* **113**, 274–284 (2004).

35. M. Koutmos, O. Kabil, J. L. Smith, R. Banerjee, Structural basis for substrate activation and regulation by cystathionine beta-synthase (CBS) domains in cystathionine β-synthase. *Proc. Natl. Acad. Sci. U.S.A.* **107**, 20958–20963 (2010).

36. T. Majtan, A. L. Pey, R. Fernández, J. A. Fernández, L. A. Martínez-Cruz, J. P. Kraus, Domain organization, catalysis and regulation of eukaryotic cystathionine beta-synthases, *PLOS ONE* **9**, e105290 (2014).

37. R. Evande, S. Ojha, R. Banerjee, Visualization of PLP-bound intermediates in hemeless variants of human cystathionine β-synthase: Evidence that lysine 119 is a general base. *Arch. Biochem. Biophys.* **427**, 188–196 (2004).

38. A. Kumar, A. Wißbrock, N. Goradia, P. Bellstedt, R. Ramachandran, D. Imhof, O. Ohlenschläger, Heme interaction of the intrinsically disordered N-terminal peptide segment of human cystathionine-β-synthase. *Sci. Rep.* **8**, 2474 (2018).

39. J. Oliveriusova, V. Kery, K. N. Maclean, J. P. Kraus, Deletion mutagenesis of human cystathionine beta-synthase—Impact on activity, oligomeric status, and *S*-adenosylmethionine regulation. *J. Biol. Chem.* **277**, 48386–48394 (2002).

40. R. Vozdek, A. Hnizda, J. Krijt, M. Kostrouchova, V. Kozich, Novel structural arrangement of nematode cystathionine β-synthases: Characterization of *Caenorhabditis elegans* CBS-1. *Biochem. J.* **443**, 535–547 (2012).

41. K.-H. Jhee, P. McPhie, E. W. Miles, Yeast cystathionine β-synthase is a pyridoxal phosphate enzyme but, unlike the human enzyme, is not a heme protein. *J. Biol. Chem.* **275**, 11541–11544 (2000).

42. G. Dixon, E. Abbott, M. Matz, Meta-analysis of the coral environmental stress response: Acropora corals show opposing responses depending on stress intensity. *Mol. Ecol.* **29**, 2855–2870 (2020).

43. R. Savary, D. J. Barshis, C. R. Voolstra, A. Cárdenas, N. R. Evensen, G. Banc-Prandi, M. Fine, A. Meibom, Fast and pervasive transcriptomic resilience and acclimation of extremely heat-tolerant coral holobionts from the northern Red Sea. *Proc. Natl. Acad. Sci. U.S.A.* **118**, e2023298118 (2021).

44. G. W. Vurture, F. J. Sedlazeck, M. Nattestad, C. J. Underwood, H. Fang, J. Gurtowski, M. C. Schatz, GenomeScope: Fast reference-free genome profiling from short reads. *Bioinformatics* **33**, 2202–2204 (2017).

45. G. Marcais, C. Kingsford, A fast, lock-free approach for efficient parallel counting of occurrences of k-mers. *Bioinformatics* **27**, 764–770 (2011).

46. A. M. Bolger, M. Lohse, B. Usadel, Trimmomatic: A flexible trimmer for Illumina sequence data. *Bioinformatics* **30**, 2114–2120 (2014).

47. L. Salmela, E. Rivals, LoRDEC: Accurate and efficient long read error correction. *Bioinformatics* **30**, 3506–3514 (2014).

48. S. Koren, B. P. Walenz, K. Berlin, J. R. Miller, N. H. Bergman, A. M. Phillippy, Canu: Scalable and accurate long-read assembly via adaptive *k*-mer weighting and repeat separation. *Genome Res.* **27**, 722–736 (2017).

49. M. J. Roach, S. A. Schmidt, A. R. Borneman, Purge haplotigs: Allelic contig reassignment for third-gen diploid genome assemblies. *BMC Bioinform.* **19**, 460 (2018).

50. B. J. Walker, T. Abeel, T. Shea, M. Priest, A. Abouelliel, S. Sakthikumar, C. A. Cuomo, Q. Zeng, J. Wortman, S. K. Young, A. M. Earl, Pilon: An integrated tool for comprehensive microbial variant detection and genome assembly improvement. *PLOS ONE* **9**, e112963 (2014).

51. H. Li, Minimap2: Pairwise alignment for nucleotide sequences. *Bioinformatics* **34**, 3094–3100 (2018).

52. H. Li, Aligning sequence reads, clone sequences and assembly contigs with BWA-MEM. arXiv:1303.3997 (2013).

53. M. Boetzer, W. Pirovano, SSPACE-longRead: Scaffolding bacterial draft genomes using long read sequence information. *BMC Bioinform.* **15**, 211 (2014).

54. S. Yeo, L. Coombe, R. L. Warren, J. Chu, I. Birol, ARCS: Scaffolding genome drafts with linked reads. *Bioinformatics* **34**, 725–731 (2018).

55. G. C. Xu, T.J. Xu, R. Zhu, Y. Zhang, S.Q. Li, H.W. Wang, J.T. Li, LR_Gapcloser: A tiling path-based gap closer that uses long reads to complete genome assembly. *Gigascience* **8**, giy157 (2019).

56. S. F. Huang, M. J. Kang, A. L. Xu, HaploMerger2: Rebuilding both haploid sub-assemblies from high-heterozygosity diploid genome assembly. *Bioinformatics* **33**, 2577–2579 (2017).

57. K. J. Hoff, A. Lomsadze, M. Borodovsky, M. Stanke, Whole-genome annotation with BRAKER. *Methods Mol. Biol.* **1962**, 65–95 (2019).

58. K. J. Hoff, S. Lange, A. Lomsadze, M. Borodovsky, M. Stanke, BRAKER1: Unsupervised RNA-Seq-based genome annotation with GeneMark-ET and AUGUSTUS. *Bioinformatics* **32**, 767–769 (2016).

59. A. Smit, R. Hubley, RepeatModeler Open-1.0 (2008-2015); www.repeatmasker.org.

60. A. Smit, R. Hubley, P. Green, RepeatMasker Open-4.0 (2013-2015); www.repeatmasker.org.

61. D. Kim, J. M. Paggi, C. Park, C. Bennett, S. L. Salzberg, Graph-based genome alignment and genotyping with HISAT2 and HISAT-genotype. *Nat. Biotechnol.* **37**, 907–915 (2019).

62. I. Cooke, H. Ying, S. Forêt, P. Bongaerts, J. M. Strugnell, O. Simakov, J. Zhang, M. A. Field, M. Rodriguez-Lanetty, S. C. Bell, D. G. Bourne, Ma. J. van Oppen, M. A. Ragan, D. J. Miller, Genomic signatures in the coral holobiont reveal host adaptations driven by Holocene climate change and reef specific symbionts. *Sci. Adv.* **6**, eabc6318 (2020).

63. Y. J. Liew, M. Aranda, C. R. Voolstra, Reefgenomics.Org—A repository for marine genomics data. *Database (Oxford)* **2016**, 1–14 (2016).

64. H. Ying, D. C. Hayward, I. Cooke, W. Wang, A. Moya, K. R. Siemering, S. Sprungala, E. E. Ball, S. Forêt, D. J. Miller, The whole-genome sequence of the coral *Acropora millepora*. *Genome Biol. Evol.* **11**, 1374–1379 (2019).

65. NCBI Resource Coordinators, Database resources of the National Center for Biotechnology Information. *Nucleic Acids Res.* **44**, D7–D19 (2016).

66. P. Jones, D. Binns, H.Y. Chang, M. Fraser, W. Li, C. McAnulla, H. McWilliam, J. Maslen, A. Mitchell, G. Nuka, S. Pesseat, A. F. Quinn, A. Sangrador-Vegas, M. Scheremetjew, S.Y. Yong, R. Lopez, S.

Hunter, InterProScan 5: Genome-scale protein function classification. *Bioinformatics* **30**, 1236–1240 (2014).

67. M. Ashburner, C. A. Ball, J. A. Blake, D. Botstein, H. Butler, J. M. Cherry, A. P. Davis, K. Dolinski, S. S. Dwight, J. T. Eppig, M. A. Harris, D. P. Hill, L. Issel-Tarver, A. Kasarskis, S. Lewis, J. C. Matese, J. E. Richardson, M. Ringwald, G. M. Rubin, G. Sherlock, Gene ontology: Tool for the unification of biology. *Nat. Genet.* **25**, 25–29 (2000).

68. M. Kanehisa, Y. Sato, K. Morishima, BlastKOALA and GhostKOALA: KEGG tools for functional characterization of genome and metagenome sequences. *J. Mol. Biol.* **428**, 726–731 (2016).

69. C. R. Voolstra, D. J. Miller, M. A. Ragan, A. Hoffmann, O. Hoegh-Guldberg, D. Bourne, E. E. Ball, H. Ying, S. Foret, S. Takahashi, K. D. Weynberg, M. van Oppen, K. Morrow, C. X. Chan, N. Rosic, W. Leggat, S. Sprungala, M. Imelfort, G. W. Tyson, K. Kassahn, P. Lundgren, R. Beeden, T. Ravasi, M. L. Berumen, E. Abel, T. Fyffe, K. M. Morrow, The ReFuGe 2020 Consortium-using "omics" approaches to explore the adaptability and resilience of coral holobionts to environmental change. *Front. Mar. Sci.* **2**, 1–8 (2015).

70. R. Cunning, R. A. Bay, P. Gillette, A. C. Baker, N. Traylor-Knowles, Comparative analysis of the *Pocillopora damicornis* genome highlights role of immune system in coral evolution. *Sci. Rep.* **8**, 16134 (2018).

71. C. R. Voolstra, Y. Li, Y. J. Liew, S. Baumgarten, D. Zoccola, J.F. Flot, S. Tambutté, D. Allemand, M. Aranda, Comparative analysis of the genomes of *Stylophora pistillata* and *Acropora digitifera* provides evidence for extensive differences between species of corals. *Sci. Rep.* **7**, 17583 (2017).

72. C. S. Wilding, N. Fletcher, E. K. Smith, P. Prentis, G. D. Weedall, Z. Stewart, The genome of the sea anemone *Actinia equina* (L.): Meiotic toolkit genes and the question of sexual reproduction. *Mar Genomics* **53**, 100753 (2020).

73. S. Baumgarten, O. Simakov, L. Y. Esherick, Y. J. Liew, E. M. Lehnert, C. T. Michell, Y. Li, E. A. Hambleton, A. Guse, M. E. Oates, J. Gough, V. M. Weis, M. Aranda, J. R. Pringle, C. R. Voolstra, The

genome of *Aiptasia*, a sea anemone model for coral symbiosis. *Proc. Natl. Acad. Sci. U.S.A.* **112**, 11893–11898 (2015).

74. N. H. Putnam, M. Srivastava, U. Hellsten, B. Dirks, J. Chapman, A. Salamov, A. Terry, H. Shapiro, E. Lindquist, V. V. Kapitonov, J. Jurka, G. Genikhovich, I. V. Grigoriev, S. M. Lucas, R. E. Steele, J. R. Finnerty, U. Technau, M. Q. Martindale, D. S. Rokhsar, Sea anemone genome reveals ancestral eumetazoan gene repertoire and genomic organization. *Science* **317**, 86–94 (2007).

75. Y. Jeon, S. G. Park, N. Lee, J. A. Weber, H.S. Kim, S.J. Hwang, S. Woo, H.M. Kim, Y. Bhak, S. Jeon, N. Lee, Y. Jo, A. Blazyte, T. Ryu, Y. S. Cho, H. Kim, J.H. Lee, H. S. Yim, J. Bhak, S. Yum, The draft genome of an Octocoral, *Dendronephthya gigantea*. *Genome Biol. Evol.* **11**, 949–953 (2019).

76. A. L. Mitchell, T. K. Attwood, P. C. Babbitt, M. Blum, P. Bork, A. Bridge, S. D. Brown, H.Y. Chang, S. el-Gebali, M. I. Fraser, J. Gough, D. R. Haft, H. Huang, I. Letunic, R. Lopez, A. Luciani, F. Madeira, A. Marchler-Bauer, H. Mi, D. A. Natale, M. Necci, G. Nuka, C. Orengo, A. P. Pandurangan, T. Paysan-Lafosse, S. Pesseat, S. C. Potter, M. A. Qureshi, N. D. Rawlings, N. Redaschi, L. J. Richardson, C. Rivoire, G. A. Salazar, A. Sangrador-Vegas, C. J. A. Sigrist, I. Sillitoe, G. G. Sutton, N. Thanki, P. D. Thomas, S. C. E. Tosatto, S.Y. Yong, R. D. Finn, InterPro in 2019: Improving coverage, classification and access to protein sequence annotations. *Nucleic Acids Res.* **47**, D351–D360 (2019).

77. B. Q. Minh, H. A. Schmidt, O. Chernomor, D. Schrempf, M. D. Woodhams, A. von Haeseler, R. Lanfear, IQ-TREE 2: New models and efficient methods for phylogenetic inference in the genomic era. *Mol. Biol. Evol.* **37**, 1530–1534 (2020).

78. D. T. Hoang, O. Chernomor, A. von Haeseler, B. Q. Minh, L. S. Vinh, UFBoot2: Improving the ultrafast bootstrap approximation. *Mol. Biol. Evol.* **35**, 518–522 (2017).

79. I. Letunic, P. Bork, Interactive tree of life (iTOL) v5: An online tool for phylogenetic tree display and annotation. *Nucleic Acids Res.* **49**, W293–W296 (2021).

80. R. Daniel Gietz, R. A. Woods, in *Methods Enzymol.*, C. Guthrie, G. R. Fink, Eds. (Academic Press, 2002), vol. 350, pp. 87–96.

81. R. Hubley, R. D. Finn, J. Clements, S. R. Eddy, T. A. Jones, W. Bao, A. F.A. Smit, T. J. Wheeler, The Dfam database of repetitive DNA families. *Nucleic Acids Res.* **44**, D81–D89 (2016).

82. W. D. Bao, K. K. Kojima, O. Kohany, Repbase update, a database of repetitive elements in eukaryotic genomes. *Mobile DNA* **6** 11 (2015).

83. M. Stanke, M. Diekhans, R. Baertsch, D. Haussler, Using native and syntenically mapped cDNA alignments to improve de novo gene finding. *Bioinformatics* **24**, 637–644 (2008).

84. M. Stanke, O. Schoffmann, B. Morgenstern, S. Waack, Gene prediction in eukaryotes with a generalized hidden Markov model that uses hints from external sources. *BMC Bioinformatics* **7**, 62 (2006).

85. L. Li, C. J. Stoeckert, D. S. Roos, OrthoMCL: Identification of ortholog groups for eukaryotic genomes. *Genome Res.* **13**, 2178–2189 (2003).

86. S. F. Altschul, W. Gish, W. Miller, E. W. Myers, D. J. Lipman, Basic local alignment search tool. *J. Mol. Biol.* **215**, 403–410 (1990).

87. The Gene Ontology Consortium, The gene ontology resource: 20 years and still going strong. *Nucleic Acids Res.* **47**, D330–D338 (2019).

88. S. Maere, K. Heymans, M. Kuiper, BiNGO: A cytoscape plugin to assess overrepresentation of gene ontology categories in biological networks. *Bioinformatics* **21**, 3448–3449 (2005).

89. P. Shannon, A. Markiel, O. Ozier, N. S. Baliga, J. T. Wang, D. Ramage, N. Amin, B. Schwikowski, T. Ideker, Cytoscape: A software environment for integrated models of biomolecular interaction networks. *Genome Res.* **13**, 2498–2504 (2003).

90. F. Supek, M. Bosnjak, N. Skunca, T. Smuc, REVIGO summarizes and visualizes long lists of gene ontology terms. *PLOS ONE* **6**, e21800 (2011).

91. R. C. Edgar, MUSCLE: Multiple sequence alignment with high accuracy and high throughput. *Nucleic Acids Res.* **32**, 1792–1797 (2004).

92. J. Castresana, Selection of conserved blocks from multiple alignments for their use in phylogenetic analysis. *Mol. Biol. Evol.* **17**, 540–552 (2000).

93. G. Talavera, J. Castresana, Improvement of phylogenies after removing divergent and ambiguously aligned blocks from protein sequence alignments. *Syst. Biol.* **56**, 564–577 (2007).

94. D. Darriba, D. Posada, A. M. Kozlov, A. Stamatakis, B. Morel, T. Flouri, ModelTest-NG: A new and scalable tool for the selection of DNA and protein evolutionary models. *Mol. Biol. Evol.* **37**, 291–294 (2020).

95. A. M. Kozlov, D. Darriba, T. Flouri, B. Morel, A. Stamatakis, RAxML-NG: A fast, scalable and user-friendly tool for maximum likelihood phylogenetic inference. *Bioinformatics* **35**, 4453–4455 (2019).

96. Z. L. Fuller, V. J. L. Mocellin, L. A. Morris, N. Cantin, J. Shepherd, L. Sarre, J. Peng, Y. Liao, J. Pickrell, P. Andolfatto, M. Matz, L. K. Bay, M. Przeworski, Population genetics of the coral *Acropora millepora*: Toward genomic prediction of bleaching. *Science* **369**, eaba4674 (2020).

97. N. I. Weisenfeld, V. Kumar, P. Shah, D. M. Church, D. B. Jaffe, Direct determination of diploid genome sequences. *Genome Res.* **27**, 757–767 (2017).

98. R. Kajitani, K. Toshimoto, H. Noguchi, A. Toyoda, Y. Ogura, M. Okuno, M. Yabana, M. Harada, E. Nagayasu, H. Maruyama, Y. Kohara, A. Fujiyama, T. Hayashi, T. Itoh, Efficient de novo assembly of highly heterozygous genomes from whole-genome shotgun short reads. *Genome Res.* **24**, 1384–1395 (2014).

99. G. Zhang, X. Fang, X. Guo, L. Li, R. Luo, F. Xu, P. Yang, L. Zhang, X. Wang, H. Qi, Z. Xiong, H. Que, Y. Xie, P. W. H. Holland, J. Paps, Y. Zhu, F. Wu, Y. Chen, J. Wang, C. Peng, J. Meng, L. Yang, J. Liu, B. Wen, N. Zhang, Z. Huang, Q. Zhu, Y. Feng, A. Mount, D. Hedgecock, Z. Xu, Y. Liu, T. Domazet-Lošo, Y. Du, X. Sun, S. Zhang, B. Liu, P. Cheng, X. Jiang, J. Li, D. Fan, W. Wang, W. Fu, T. Wang, B. Wang, J. Zhang, Z. Peng, Y. Li, N. Li, J. Wang, M. Chen, Y. He, F. Tan, X. Song, Q. Zheng, R. Huang, H. Yang, X. Du, L. Chen, M. Yang, P. M. Gaffney, S. Wang, L. Luo, Z. She, Y. Ming, W. Huang, S. Zhang, B. Huang, Y. Zhang, T. Qu, P. Ni, G. Miao, J. Wang, Q. Wang, C. E. W. Steinberg,

H. Wang, N. Li, L. Qian, G. Zhang, Y. Li, H. Yang, X. Liu, J. Wang, Y. Yin, J. Wang, The oyster genome reveals stress adaptation and complexity of shell formation. *Nature* **490**, 49–54 (2012).

100. J. T. Simpson, Exploring genome characteristics and sequence quality without a reference. *Bioinformatics* **30**, 1228–1235 (2014).

101. K. I. Savage, D. P. Harkin, BRCA1, a 'complex' protein involved in the maintenance of genomic stability. *FEBS J.* **282**, 630–646 (2015).

102. S. Kim, W. Zeng, S. Bernard, J. Liao, M. Venkateshwaran, J.M. Ane, Y. Jiang, Publisher correction: $Ca2^{+}$regulated Ca^{2+} channels with an RCK gating ring control plant symbiotic associations. *Nat. Commun.* **10**, 3703–4607 (2019).

103. Y. P. Zhang, E. L. Pohlmann, J. Serate, M. C. Conrad, G. P. Roberts, Mutagenesis and functional characterization of the four domains of GlnD, a bifunctional nitrogen sensor protein. *J. Bacteriol.* **192**, 2711–2721 (2010).

104. E. Garcia, S. G. Rhee, Cascade control of escherichia-coli glutamine-synthetase - purification and properties of Pii uridylyltransferase and uridylyl-removing enzyme. *J. Biol. Chem.* **258**, 2246–2253 (1983).

105. R. Vozdek, A. Hnízda, J. Krijt, L. Šerá, V. Kožich, Biochemical properties of nematode O-acetylserine(thiol)lyase paralogs imply their distinct roles in hydrogen sulfide homeostasis. *Biochim. Biophys. Acta* **1834**, 2691–2701 (2013).

106. D. K. Ma, R. Vozdek, N. Bhatla, H. CYSL-1 interacts with the O2-sensing hydroxylase EGL-9 to promote H2S-modulated hypoxia-induced behavioral plasticity in C. elegans. *Neuron* **73**, 925–940 (2012).

107. M. W. Budde, M. B. Roth, The response of caenorhabditis elegans to hydrogen sulfide and hydrogen cyanide. *Genetics* **189**, 521–532 (2011).
